# Direct cellular contact by *Porphyromonas gingivalis* suppresses nociceptor excitability and induces analgesia during inflammation

**DOI:** 10.1126/sciadv.aee8088

**Published:** 2026-09-25

**Authors:** Sena Chung, Doyun Kim, Byeonggeon Koh, Yeon Kyeong Ko, Kihwan Lee, Hayun Kim, Hyun Young Kim, Manda Yu, Ozge Erdogan, Larissa Staurengo-Ferrari, Isaac M. Chiu, Mary E. Davey, Youngnim Choi, Seog Bae Oh

**Affiliations:** ^1^ Department of Brain and Cognitive Sciences, College of Natural Sciences, Seoul National University; Seoul, 08826, Republic of Korea.; ^2^ Department of Neurobiology and Physiology, School of Dentistry and Dental Research Institute, Seoul National University; Seoul, 03080, Republic of Korea.; ^3^ Department of Immunology and Molecular Microbiology, School of Dentistry and Dental Research Institute, Seoul National University; Seoul, 03080, Republic of Korea.; ^4^ Interdisciplinary Program in Neuroscience, College of Natural Sciences, Seoul National University; Seoul, 08826, Republic of Korea.; ^5^ Department of Microbiology, ADA Forsyth Institute; Somerville, MA 02143, USA.; ^6^ Department of Restorative Dentistry and Biomaterial Sciences, Harvard School of Dental Medicine; Boston, MA 02115, USA.; ^7^ Department of Immunology, Blavatnik Institute, Harvard Medical School; Boston, MA 02115, USA.

## Abstract

Pain often accompanies tissue injury and inflammation. However, in certain inflammatory contexts like periodontitis, nociceptive signaling is paradoxically suppressed. The mechanisms underlying this analgesic state remain poorly understood. Here, we investigated whether and how *Porphyromonas gingivalis* (Pg) interacts with sensory neurons to induce analgesia in models of inflammatory pain. Exposure to live Pg reversed inflammatory thermal and mechanical hypersensitivity without altering local inflammation or nerve innervation. This effect required direct physical contact mediated in part by an integrin α_5_-FimA interface and gingipain protease activity and was not reproduced by heat-killed bacteria, protease-deficient mutants, or Pg-derived extracellular vesicles. At the neuronal level, Pg contact induced membrane hyperpolarization, reduced excitability of sensory neurons, and suppressed release of the nociceptive neuropeptide calcitonin gene-related peptide (CGRP). Proteomic and functional analyses identified downregulation of Fxyd2, a regulatory subunit of the Na^+^/K^+^-ATPase, linking altered ionic homeostasis to nociceptor silencing. These findings define a contact-dependent mechanism that actively suppresses peripheral pain signaling during inflammation.

## INTRODUCTION

Pain is a fundamental protective response to tissue injury and inflammation, alerting organisms to potential harm and promoting avoidance behaviors (*1*, *2*). In most inflammatory conditions, tissue damage leads to robust activation and sensitization of nociceptive sensory neurons, resulting in pain hypersensitivity (*3*, *4*). However, clinical and experimental observations increasingly indicate that pain does not always scale proportionally with the degree of inflammation or tissue destruction (*2*). In specific pathological contexts, a striking dissociation between tissue injury and pain can be observed, suggesting the existence of active pain-suppressive mechanisms (*5*, *6*). Well-established examples include *Mycobacterium ulcerans* infection, in which the secreted lipid-like toxin mycolactone induces analgesia despite formation of skin ulcers with extensive tissue necrosis (*7*, *8*), and *Bacillus anthracis* infection, in which anthrax edema toxin modulates nociceptor signaling and blunt pain perception (*9*).

Despite these observations, current models of inflammatory pain primarily emphasize pro-nociceptive mediators, including cytokines, prostaglandins, and neurotrophic factors, that enhance sensory neuron excitability (*10*). While these frameworks account for pain sensitization, they offer limited insight into pathological contexts in which pain is actively suppressed despite ongoing inflammation, as observed in toxin-mediated analgesia. Notably, all well-characterized examples of bacterial analgesia to date rely on diffusible toxins that broadly impair neuronal function, leaving unresolved whether pain suppression can arise through alternative, non-toxin-based mechanisms.

Periodontitis represents a clinically prevalent example of pain-pathology dissociation. Despite substantial tissue destruction, bleeding, and bone resorption, pain is usually mild or absent (*11*–*13*), in marked contrast to other infections such as *Streptococcus pyogenes*-induced skin and soft tissue infections, where pain is an early and prominent symptom (*14*). The absence of pain in periodontitis often delays diagnosis and treatment, contributing not only to irreversible tooth loss but also to sustained local inflammation. Although the immunopathogenesis of periodontitis has been extensively characterized, the mechanisms underlying its reduced pain hypersensitivity remain poorly defined.

A central feature of periodontitis is infection with *Porphyromonas gingivalis* (Pg), a key pathobiont that drives subgingival dysbiosis and chronic periodontal inflammation (*15*). Pg expresses a distinctive repertoire of virulence factors, including gingipains, fimbriae, and atypical lipopolysaccharide (LPS) structures, that enable immune evasion and persistence within inflamed tissues (*16*). Although the immunopathogenic roles of these virulence factors have been studied in detail, whether Pg can directly influence sensory neuron function and nociceptive signaling, rather than acting indirectly through immune or soluble pathways, remains unknown. Given that local microbial interactions can have consequences beyond the site of infection, understanding how Pg engages sensory neurons at the tissue level may inform broader effects of this pathobiont.

Nociceptor excitability determines how inflammatory signals are encoded as pain, and even subtle modulation of this excitability has the potential to reshape disease perception and progression. However, whether bacterial pathogens can modulate nociceptor function to suppress pain during ongoing inflammation has not been established. Consistent with emerging evidence that bacteria can interact with nociceptive neurons, this study investigates whether and how Pg influences peripheral nociceptor excitability under inflammatory conditions. By identifying the molecular and cellular mechanisms underlying this interaction, we aim to elucidate a previously unrecognized mode of bacterial modulation of pain signaling.

## RESULTS

### Pg selectively suppresses inflammatory pain in peripheral models

**Fig. 1.**
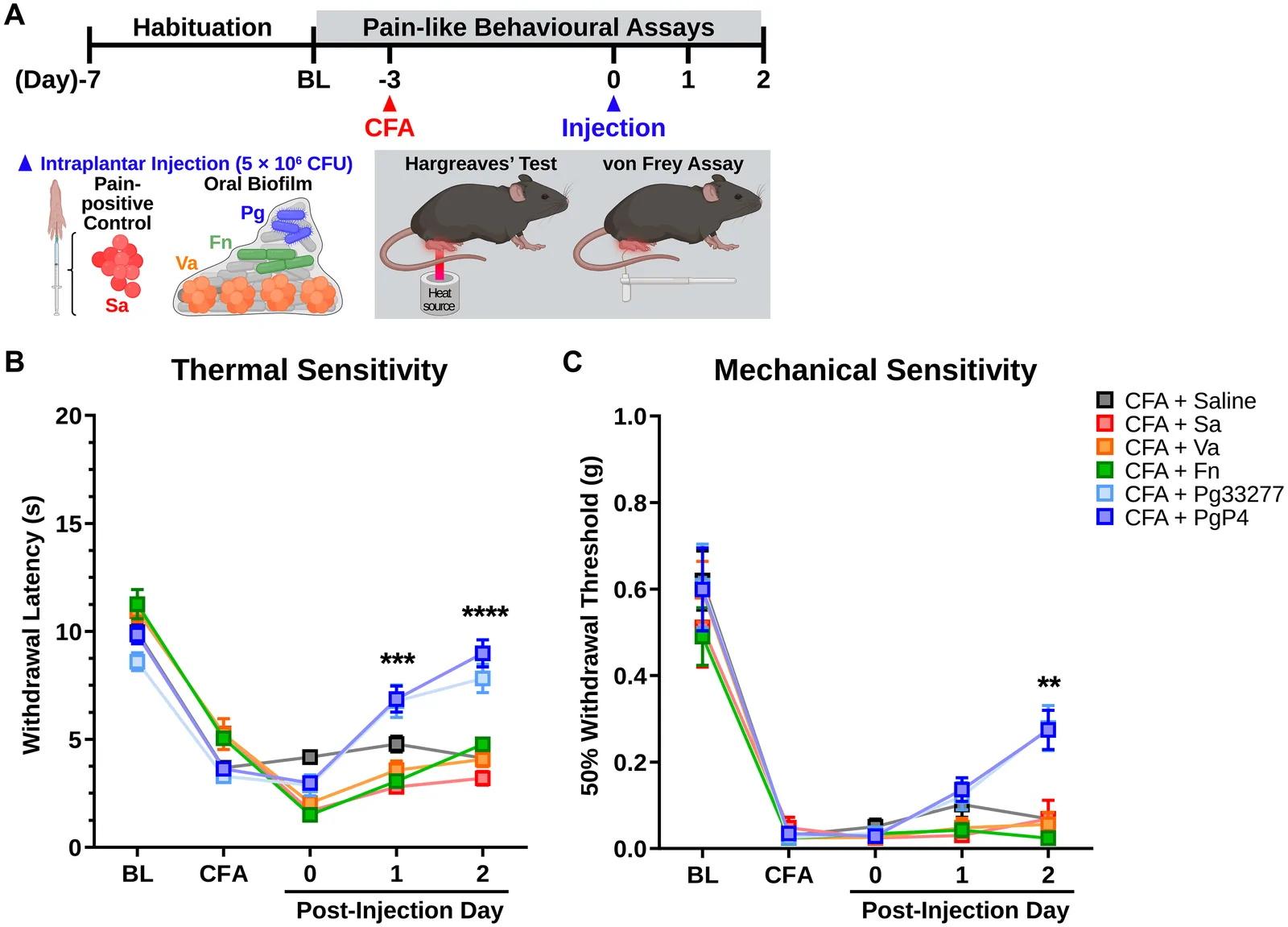
Pg selectively suppresses inflammatory pain in a chronic inflammatory model. (**A**) Experiment timeline for hind paw pain-like behavioral assays after an intraplantar injection of candidate bacteria in the CFA-induced inflammatory pain model (created in BioRender. Chung, S. https://BioRender.com/t0j5lk2). (**B**) Thermal sensitivity measured by Hargreaves’ test at CFA (3 hours post-CFA/3 days prior to bacterial injection), 0 (3 hours post-injection), 1, and 2 days after intraplantar injection of saline (*n* = 23), Sa (*n* = 9), Va (*n* = 9), Fn (*n* = 9), Pg33277 (*n* = 10), or PgP4 (*n* = 20) (*P* = 0.0003 for PgP4 and *P* = 0.0144 for Pg33277 vs. saline at post-injection day (PID) 1, *P* < 0.0001 for both Pg33277 and PgP4 vs. saline at PID2). (**C**) Mechanical sensitivity measured by von Frey assay at CFA (3 hours post-CFA/3 days prior to bacterial injection), 0 (3 hours post-injection), 1, and 2 days after intraplantar injection of saline (*n* = 19), Sa (*n* = 9), Va (*n* = 9), Fn (*n* = 9), Pg33277 (*n* = 13), or PgP4 (*n* = 22) (*P* = 0.0027 for PgP4 and *P* = 0.013 for Pg33277 vs. saline at PID2). Two-way ANOVA with Bonferroni’s multiple comparisons test in (B) and (C). **P < 0.01, ***P < 0.001, ****P < 0.0001. Data are presented as mean ± S.E.M. BL, baseline; CFA, complete Freund’s adjuvant; Sa, *Staphylococcus aureus*; Va, *Veillonella atypica*; Fn, *Fusobacterium nucleatum*; Pg33277, *Porphyromonas gingivalis* ATCC #33277 strain; PgP4, *P. gingivalis* KUMC-P4 strain (clinical isolate).

To determine whether *P. gingivalis* (Pg) alters pain-related behaviors under inflammatory conditions, we first compared representative oral bacterial species based on their ecological and pathogenic roles within the oral biofilm. *Veillonella atypica* (Va) served as a commensal control, whereas *Fusobacterium nucleatum* subsp. *nucleatum* (Fn) was included as a bridging species that facilitates Pg colonization and promotes gingival inflammation through pro-inflammatory cytokine induction (*17*, *18*). Pg itself—a key pathobiont (*19*)—was represented by two strains: the bacterial type strain ATCC #33277 (Pg33277) and the clinical isolate KUMC-P4 (PgP4) from a periodontitis patient (*20*). *Staphylococcus aureus* (Sa), a pathogen known to evoke strong pain responses (*21*), was included as a positive control. Persistent inflammation was induced by intraplantar injection of complete Freund’s adjuvant (CFA), followed three days later by bacterial inoculation (Fig. 1A). Bacteria were injected at 5 × 10^6^ CFU in 20 μl, a dose selected from previously established intraplantar bacterial infection and sensory neuron-bacteria interaction models to provide local bacterial exposure without overt tissue necrosis or systemic toxicity (*14*, *21*, *22*). Both Pg33277 and PgP4 significantly reversed the CFA-induced decreases in thermal withdrawal latency and 50% mechanical withdrawal threshold at post-injection day (PID) 2, whereas Va, Fn, and Sa had no effect (Fig. 1, B and C), indicating that analgesia is specifically associated with Pg infection under inflammatory conditions.

To gain insight into how Pg induces analgesia, we next examined pain-related behaviors under naïve conditions to determine whether Pg alters basal nociceptive sensitivity or elicits pain in the absence of inflammation. In the naïve hind paw, neither Pg33277, PgP4, Fn, nor Va altered spontaneous pain-like behavior at 6 hours after injection, whereas Sa tended to increase licking and biting time (fig. S1A), showing that Pg did not evoke pain behavior. At 7 hours post-injection, immediately after the spontaneous pain assessment, hind paw thickness was increased by Sa and Fn, but not by the commensal Va (fig. S1B). Pg33277 and PgP4 tended to increase hind paw thickness, likely reflecting transient local swelling. In preliminary dose comparison experiments, a higher PgP4 dose (1 × 10^8^ CFU in 20 μl) did not induce spontaneous pain-like behavior but produced marked paw swelling (fig. S1, A and B), supporting the use of 5 × 10^6^ CFU for subsequent behavioral experiments to avoid local swelling while assessing bacterial modulation of inflammatory pain. Because the non-Pg bacteria did not reverse CFA-induced hypersensitivity in Fig. 1, the evoked naïve assays were focused on Pg33277 and PgP4 to determine whether the analgesia-associated Pg strains alter baseline thermal or mechanical sensitivity in the absence of inflammation. Evoked behavioral assays revealed a transient increase in thermal sensitivity at PID0 that resolved by PID1, with no change in mechanical sensitivity (fig. S1, C and D); by PID2, paw thickness was not different from saline (fig. S1E). Together, these results indicate that Pg does not cause persistent pain behavior or sustained inflammation under naïve conditions.

Consistent with these behavioral observations, hind paw inflammatory cell density and interleukin-1β (IL-1β) levels at PID2 showed no significant differences between saline- and PgP4-treated groups in either the naïve or CFA condition (fig. S1, F to H), suggesting that Pg infection does not alter the degree of inflammation. Likewise, intra-epidermal nerve fiber (IENF) density in hind paw skin was unchanged between groups (fig. S1, I and J), indicating that nerve degeneration is not a contributing factor. Overall, these findings suggest that Pg-induced analgesia is not attributable to modulation of inflammatory responses or peripheral nerve degeneration. We therefore next sought to identify bacterial virulence factors that mediate this effect.

### Gingipain activity is required but not sufficient for Pg-induced analgesia

**Fig. 2.**
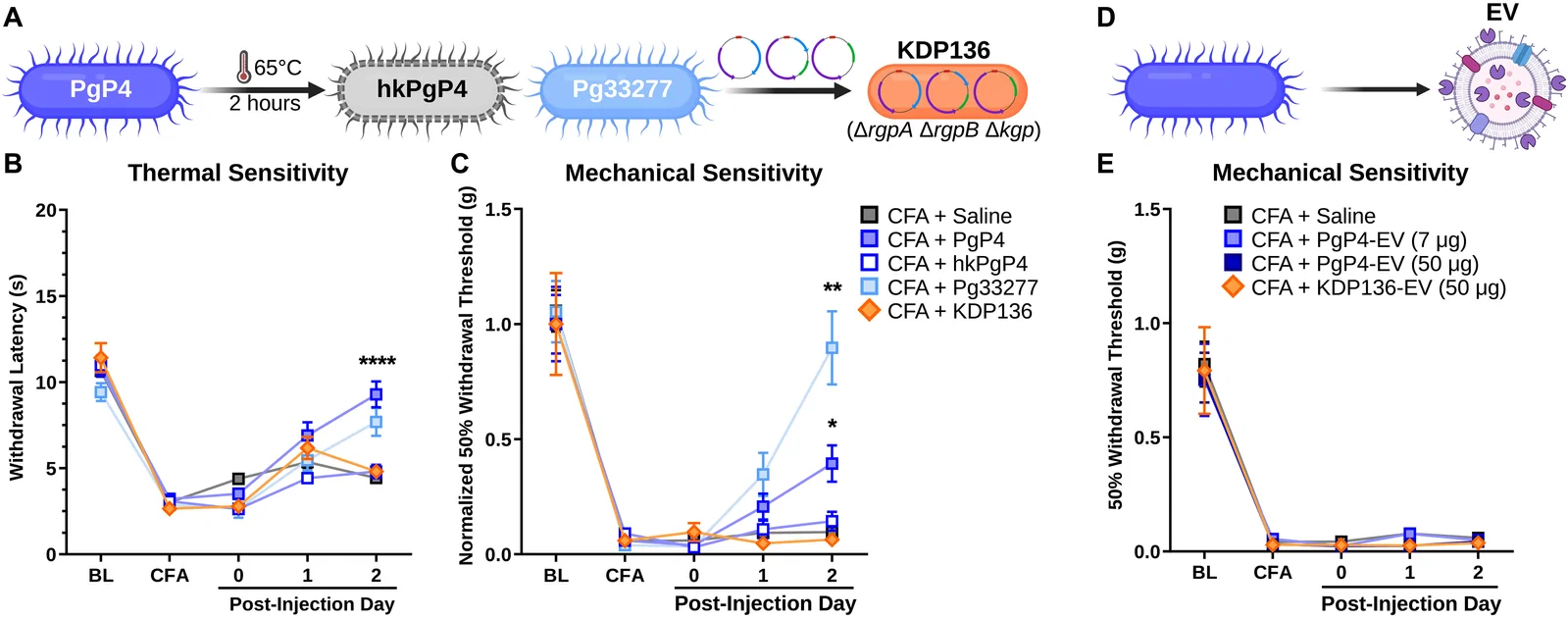
Gingipains are necessary, but not sufficient, to induce analgesia. (**A**) Schematic overview of bacterial preparations used in the experiments: PgP4 (clinical isolate), heat-killed PgP4 (hkPgP4), Pg33277 (type strain), and KDP136 (gingipain-null mutant derived from Pg33277). (**B**) Thermal sensitivity measured by Hargreaves’ test at CFA (3 hours post-CFA & 3 days prior to bacterial injection), 0 (3 hours post-injection), 1, and 2 days after intraplantar injection of saline (*n* = 28), PgP4 (*n* = 15), hkPgP4 (*n* = 12), Pg33277 (*n* = 10), or KDP136 (*n* = 10) (*P* < 0.0001 for both Pg33277 and PgP4 vs. saline at post-injection day 2). (**C**) Mechanical sensitivity measured by von Frey assay at CFA (3 hours post-CFA & 3 days prior to bacterial injection), 0 (3 hours post-injection), 1, and 2 days after intraplantar injection of saline (*n* = 27), PgP4 (*n* = 14), hkPgP4 (*n* = 12), Pg33277 (*n* = 10), or KDP136 (*n* = 10) (*P* = 0.0097 for Pg33277 and *P* = 0.0391 for PgP4 vs. saline at post-injection day 2). (**D**) Extracellular vesicles (EVs) isolated from PgP4 or gingipain-null mutant KDP136. (**E**) Mechanical sensitivity measured by von Frey assay at CFA (3 hours post-CFA & 3 days prior to bacterial injection), 0 (3 hours post-injection), 1, and 2 days after intraplantar injection of saline (*n* = 11), PgP4-EV (7 μg) (*n* = 6), PgP4-EV (50 μg) (*n* = 5), or KDP136-EV (50 μg) (*n* = 5). Schematics created in BioRender. Chung, S. https://BioRender.com/33vnn9w. Two-way ANOVA with Bonferroni’s multiple comparisons test in (B), (C), and (E). Only significant comparisons are indicated. *P < 0.05, **P < 0.01, ****P < 0.0001. Data are presented as mean ± S.E.M. CFA, complete Freund’s adjuvant; Pg33277, *Porphyromonas gingivalis* ATCC #33277 strain; PgP4, *P. gingivalis* KUMC-P4 strain (clinical isolate); hkPgP4, heat-killed PgP4; KDP136, gingipain-null mutant of Pg33277; EV, extracellular vesicles.

To identify bacterial components that contribute to Pg-induced modulation of inflammatory pain, we first examined whether the analgesic effect observed with live Pg was preserved after bacterial heat inactivation (Fig. 2A). In the CFA model, administration of heat-killed PgP4 (hkPgP4) failed to reproduce the analgesic effect seen with live PgP4 (Fig. 2, B and C), indicating that heat-sensitive bacterial components are required for the observed pain modulation. Based on this result, we next examined heat-labile virulence factors expressed by Pg, among which gingipains are a well-characterized family of cysteine proteases with established roles in Pg pathogenicity and host interaction. To evaluate their contribution, we tested a gingipain-null mutant strain (KDP136), generated from the parent Pg33277 strain, using the same CFA model (Fig. 2A). Similar to hkPgP4, KDP136 failed to reverse CFA-induced pain hypersensitivity, demonstrating that gingipain activity is required for Pg-induced analgesia (Fig. 2, B and C). We then asked whether gingipain activity alone was sufficient to mediate this effect. Administration of Pg-derived extracellular vesicles (EVs), which are enriched in gingipains (*23*), did not alter CFA-induced mechanical hypersensitivity when administered at low (7 μg; net protein weight extracted from 5 × 10^6^ CFU bacteria) or high (50 μg) doses (Fig. 2, D and E). Notably, EVs isolated from the gingipain-null KDP136 strain likewise failed to produce analgesic effects (Fig. 2E), indicating that gingipain-containing EVs are not sufficient to recapitulate the analgesic phenotype observed with live Pg. Together, these results indicate that while gingipain activity is necessary, it is not sufficient on its own to account for Pg-induced analgesia, suggesting that additional bacterial components are required for this pain-modulating effect. These findings prompted us to next investigate whether Pg directly modulates sensory neuron function.

Bacterial factors can affect neurons either by directly activating membrane receptors and ion channels or by engaging slower transcriptional and functional reprogramming. To distinguish between these possibilities, we examined the effects of Pg on cultured dorsal root ganglion (DRG) neurons. For in vitro functional assays, Pg was applied at 5 × 10^7^ CFU in 200 μl, corresponding to the same working concentration used for in vivo. Acute bath application of PgP4 (2.5 × 10^8^ CFU/ml) elicited no immediate calcium responses in DRG neurons (fig. S2A), consistent with the absence of acute pain behaviors in vivo and indicating that Pg does not directly excite nociceptors. Notably, Pg-induced analgesia emerged only after one to two days in vivo (Fig. 1, B and C), reinforcing that the effect is not mediated by rapid neuronal activation. We next tested whether Pg modulates neuronal sensitization under inflammatory conditions. Six-hour co-cultures of DRG neurons with PgP4 did not change Ca^2+^ responses to capsaicin or to an inflammatory mediator cocktail (bradykinin, prostaglandin E_2_, and histamine) compared with PBS-treated controls (fig. S2, B and C). This differs from *Escherichia coli* LPS, which sensitizes TRPV1, enhances capsaicin-induced Ca^2+^ responses (*24*, *25*), and increases prostaglandin E_2_ production (*26*, *27*). These data suggest that Pg neither activates nor sensitizes DRG neurons via canonical receptor- or channel-mediated pathways, leading us to explore alternative mechanisms by which Pg may influence neuronal function.

Accordingly, we profiled transcriptional changes in DRG neurons after 6 hours of Pg co-culture using a signaling pathway-focused qPCR array. Genes associated with NF-κB and JAK-STAT pathways showed modest upregulation (fig. S2D), reflecting a general host response to bacterial exposure rather than a specific pro-nociceptive program (*28*, *29*). These findings indicate that DRG neurons sensed Pg exposure but did not undergo acute calcium activation or inflammatory sensitization (fig. S2, A to D). In addition, TUNEL staining revealed no increase in neuronal cell death following co-culture with Pg33277 or PgP4 (fig. S2E), consistent with the absence of nerve fiber degeneration observed in vivo (fig. S1, I and J). Together, these results suggest that Pg engages sensory neurons without eliciting acute activation, inflammatory sensitization, or cytotoxicity.

### Gingipain-dependent physical contact enables Pg-nociceptor interaction

Because Pg-induced analgesia required live Pg but was not recapitulated by hkPg or Pg-derived EVs, diffusible bacterial products alone appeared insufficient to explain Pg-induced neuronal modulation. We therefore considered whether intact Pg suppresses sensory neurons through direct physical association with neuronal membranes. This possibility was particularly relevant because Pg fimbriae mediate host-cell adhesion, while gingipains regulate fimbrial maturation in addition to their protease activity (*30*). Thus, the loss of analgesic activity in the gingipain-null mutant could reflect impaired protease activity, defective adhesion-dependent neuronal engagement, or both. Based on this reasoning, we next examined whether Pg physically associates with DRG neurons in a gingipain-dependent manner.

**Fig. 3.**
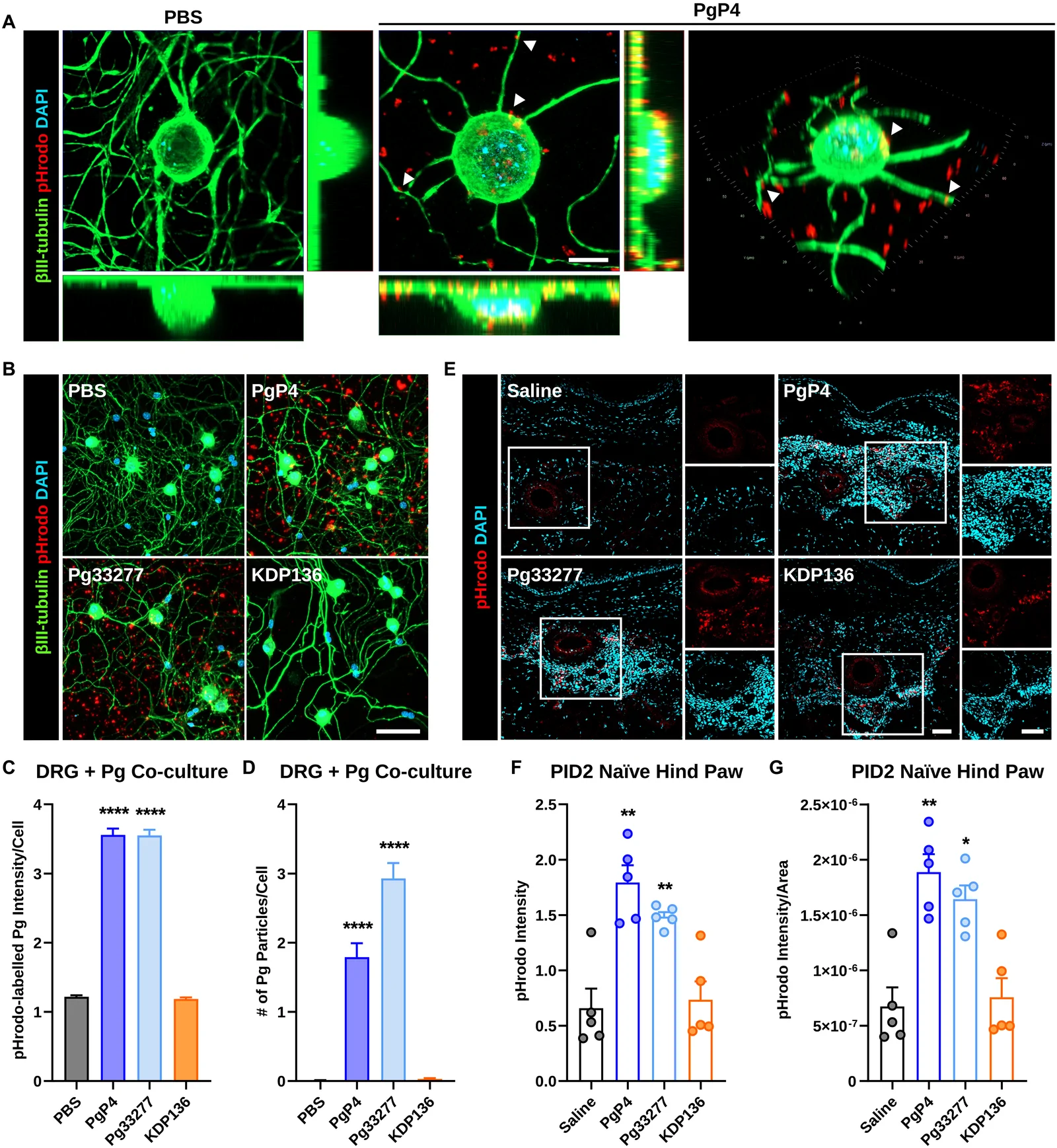
Direct physical interactions between Pg and DRG neurons require gingipains. (**A**) Co-localization of pHrodo-labelled PgP4 (indicated with white arrowheads) with βIII-tubulin- and DAPI-stained DRG neurons after 6 hours of co-culture (left). 3D reconstruction (right). Scale bar: 10 μm. (**B**) Representative images of pHrodo-labelled Pg33277, PgP4, or KDP136 with βIII-tubulin- and DAPI-stained DRG neurons after 6 hours of co-culture from three independent experiments. Scale bar: 50 μm. (**C**) Quantification of pHrodo intensity/βIII-tubulin^+^ DAPI^+^ cells in PBS (*n* = 267), PgP4 (*n* = 255), Pg33277 (*n* = 168), or KDP136 (*n* = 227) co-culture for 6 hours (*P* < 0.0001 for both PgP4 and Pg33277). One random image/group was imaged in each of three independent experiments and analyzed. (**D**) Quantification of the number of pHrodo^+^ particles/βIII-tubulin^+^ DAPI^+^ cells in PBS (*n* = 268), PgP4 (*n* = 256), Pg33277 (*n* = 168), or KDP136 (*n* = 225) co-culture for 6 hours (*P* < 0.0001 for both PgP4 and Pg33277). One random image/group was imaged in each of three independent experiments and analyzed. (**E**) Representative images of DAPI-stained naïve hind paw skin sections 2 days after pHrodo-labelled PgP4, Pg33277, or KDP136 injection. Boxed areas are magnified on the right. Scale bars: 50 μm in both full and magnified images. (**F**) Quantification of pHrodo intensity/hind paw skin sections (*n* = 5/group) (*P* = 0.0079 for both PgP4 and Pg33277). (**G**) Quantification of pHrodo intensity/hind paw skin section area (*n* = 5/group) (*P* = 0.0079 for PgP4 and *P* = 0.0159 for Pg33277). Pairwise Mann-Whitney tests for pre-specified comparisons against PBS or saline controls in (C), (D), (F), and (G). *P < 0.05, **P < 0.01, ****P < 0.0001. Data are presented as mean ± S.E.M. PgP4, *Porphyromonas gingivalis* KUMC-P4 strain (clinical isolate); Pg33277, *P. gingivalis* ATCC #33277 strain; KDP136, gingipain-null mutant of Pg33277.

To visualize Pg-neuron interactions, we fluorescently labeled Pg with pHrodo and co-cultured with DRG neurons. Confocal immunocytochemistry (ICC) revealed co-localization of Pg signals with βIII-tubulin-positive (βIII-tubulin^+^) neuronal somata and neurites, and 3D-reconstruction confirmed close apposition of Pg to neuronal membranes (Fig. 3A). Co-culture with the gingipain-null mutant KDP136 demonstrated that these interactions depend on gingipain expression. The pHrodo fluorescence intensity and the number of Pg particles associated with neurons were significantly higher in PgP4 and Pg33277 groups than in KDP136 or PBS controls (Fig. 3, B to D). Unlike wild-type (WT) Pg strains, KDP136 showed impaired neuronal association, consistent with defective fimbrial maturation. Transmission electron microscopy (TEM) further showed that KDP136 lacks fimbriae, which are required for adhesion and internalization (fig. S3). To verify these findings in vivo, pHrodo-labeled bacteria were injected into the hind paw skin. Consistent with the in vitro observations, fluorescence intensity at the injection site was markedly higher for WT strains than for KDP136 (Fig. 3, E to G), supporting the role of gingipains in bacterial adhesion and tissue persistence. Together, these findings demonstrate that gingipain-dependent physical contact represents a prerequisite for Pg-neuron interaction both in vitro and in vivo, supporting a contact-dependent mechanism of action. Unlike toxin-mediated analgesia, which can occur independently of physical proximity, Pg required gingipain-dependent physical contact to engage nociceptors.

### Pg contact hyperpolarizes nociceptors, reduces excitability, and suppresses CGRP release

**Fig. 4.**
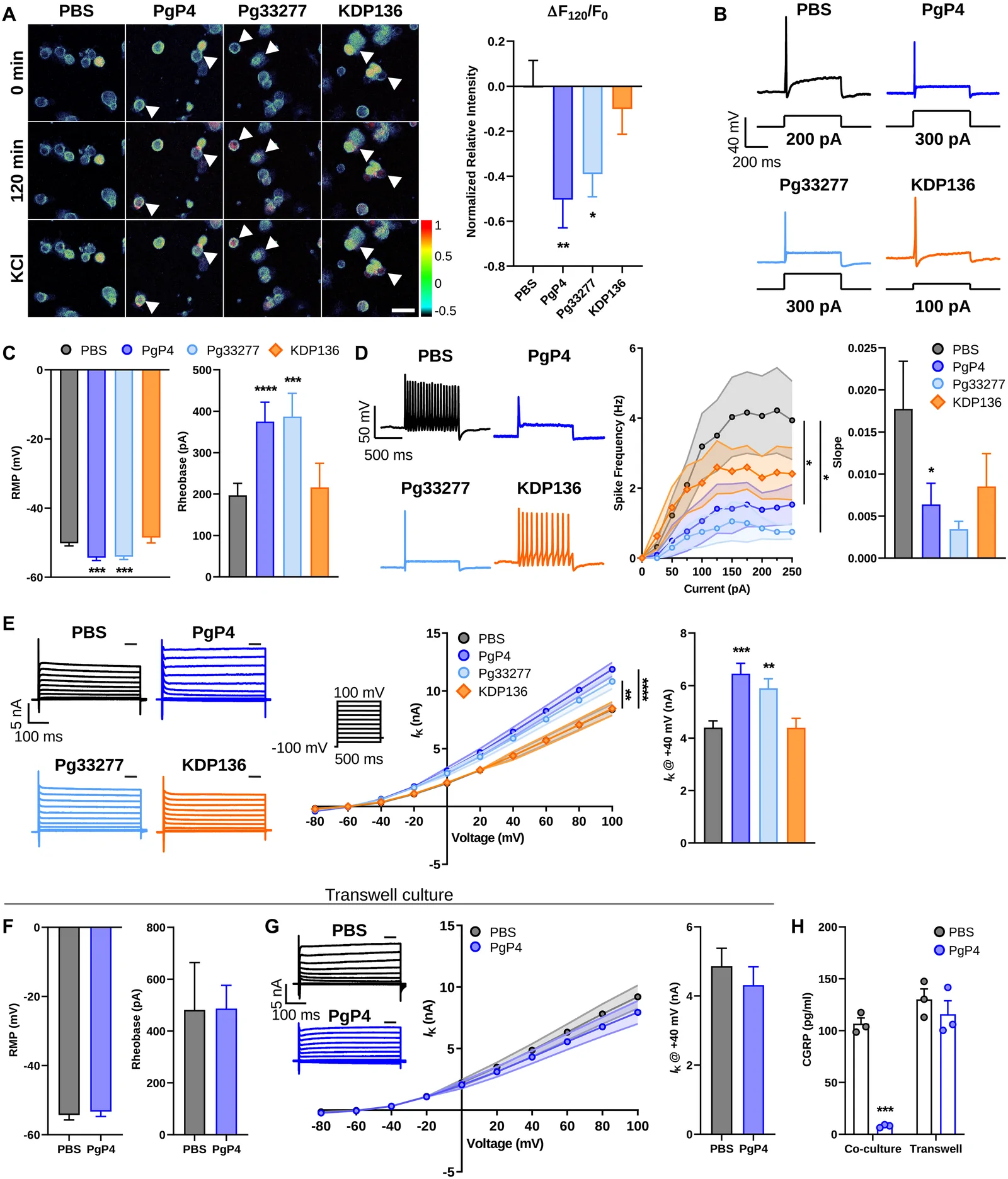
Pg contact hyperpolarizes DRG neurons and lowers excitability with increased K^+^ conductance and decreased CGRP release. (**A**) FluoVolt membrane-potential imaging and 120-min quantification in DRG neurons exposed to pHrodo-PgP4, Pg33277, or KDP136, or PBS (*n* = 80/50/48/50 for PBS/PgP4/Pg33277/KDP136; two independent experiments; *P* = 0.0024 for PgP4, *P* = 0.0118 for Pg33277). Arrowheads indicate pHrodo and FluoVolt co-localization. Scale bar: 50 μm. (**B** to **E**) Whole-cell recordings after 6-hour co-culture showing rheobase-evoked action potentials (B), RMP/rheobase (C; *n* = 77/53/62/20; *P* = 0.0024 for PgP4, *P* = 0.0118 for Pg33277), spike frequency/slope (D; *n* = 32/34/20/27; *P* = 0.0320 for PgP4 & *P* = 0.0251 for Pg33277 in frequency; *P* = 0.0444 for PgP4 in slope), and *I*_K_ (E; *n* = 24/18/18/20; *P* < 0.0001 for PgP4 & *P* = 0.0017 for Pg33277 in *I*-*V*; *P* = 0.0001 for PgP4 & *P* = 0.0012 for Pg33277 at +40 mV). (**F** and **G**) RMP/rheobase (F) and *I*_K_ (G) after transwell culture with PBS or PgP4 (*n* = 13/17; two independent experiments). (**H**) CGRP levels in co-culture or transwell supernatants after 6 hours (*n* = 3/group; two independent experiments). Electrophysiology in (B to E) used five independent experiments. Pairwise Mann-Whitney tests in (A), (C), (D, slope), (E, *I*_K_ at +40 mV), (F), and (G, *I*_K_ at +40 mV). Two-way ANOVA/Bonferroni’s tests in (D), (E), (G), and (H). Only significant comparisons are indicated. *P < 0.05, **P < 0.01, ***P < 0.001, ****P < 0.0001. Data are presented as mean ± S.E.M.

We next examined the functional consequences of Pg-neuron contact. Using the voltage-sensitive dye FluoVolt, we observed a significant decrease in fluorescence after 2 hours of co-culture with PgP4 or Pg33277, indicating membrane hyperpolarization relative to PBS and KDP136 (Fig. 4A). To further characterize changes in intrinsic excitability, whole-cell patch clamp recordings were conducted after 6 hours of co-culture. PgP4- and Pg33277-treated neurons exhibited more negative resting membrane potential (RMP) values (−54.35 ± 0.80 and −54.04 ± 0.78 mV) and increased rheobase (374.9 ± 47.07 and 387 ± 56.37 pA) compared with PBS (−50.14 ± 0.77 mV, 197 ± 28.65 pA) and KDP136 (−48.50 ± 1.58 mV, 216 ± 58.31 pA) groups (Fig. 4, B and C).

Action potential firing was quantified by step current injections (0–250 pA). Both Pg strains significantly decreased spike frequency and the slope of the frequency-current relationship relative to KDP136 and PBS controls (Fig. 4D), confirming decreased neuronal excitability upon Pg co-culture. These findings demonstrate that Pg contact directly attenuates the excitability of DRG neurons through gingipain-dependent mechanisms. To determine the ionic basis of these changes, voltage clamp recordings were used to measure potassium currents (*I*_K_). Current-voltage (*I*-*V*) relationships revealed significantly larger outward K^+^ currents in PgP4 and Pg33277 groups (6.45 ± 0.40 and 5.90 ± 0.36 nA at +40 mV) than in PBS (4.40 ± 0.26 nA) or KDP136 (4.39 ± 0.36 nA) groups (Fig. 4E). When direct contact between Pg and neurons was prevented by transwell cultures, RMP, rheobase, and *I*_K_ amplitude were indistinguishable between PgP4 and PBS conditions (Fig. 4, F and G), confirming that the electrophysiological effects require physical interaction rather than soluble factors.

Given that hyperpolarization is often associated with reduced neuropeptide release (*31*, *32*), we next examined the impact of Pg on calcitonin gene-related peptide (CGRP), a nociceptor-derived neuropeptide released from peripheral terminals implicated in pain and neuro-immune signaling, including neurogenic inflammation (*14*, *33*, *34*). PgP4 significantly decreased CGRP release from DRG neurons after 6 hours of co-culture (Fig. 4H), whereas this suppression was absent in transwell conditions, demonstrating a requirement for direct contact. Among all bacterial species tested, only PgP4—but not Sa, Va, or Fn—significantly reduced CGRP secretion (fig. S4A). Furthermore, PgP4 suppressed CGRP release even under depolarizing conditions induced by 50 mM extracellular K^+^ (fig. S4B). Consistent with these in vitro findings, PgP4 injection in vivo significantly reduced CGRP levels in hind paw tissues at PID2 (fig. S4, C and D). Collectively, these results establish a functional link between Pg-neuron contact, reduced excitability, and suppressed nociceptor output.

### Integrin α_5_-FimA interaction mediates neuron-Pg contact to induce neuronal hypoexcitability

**Fig. 5.**
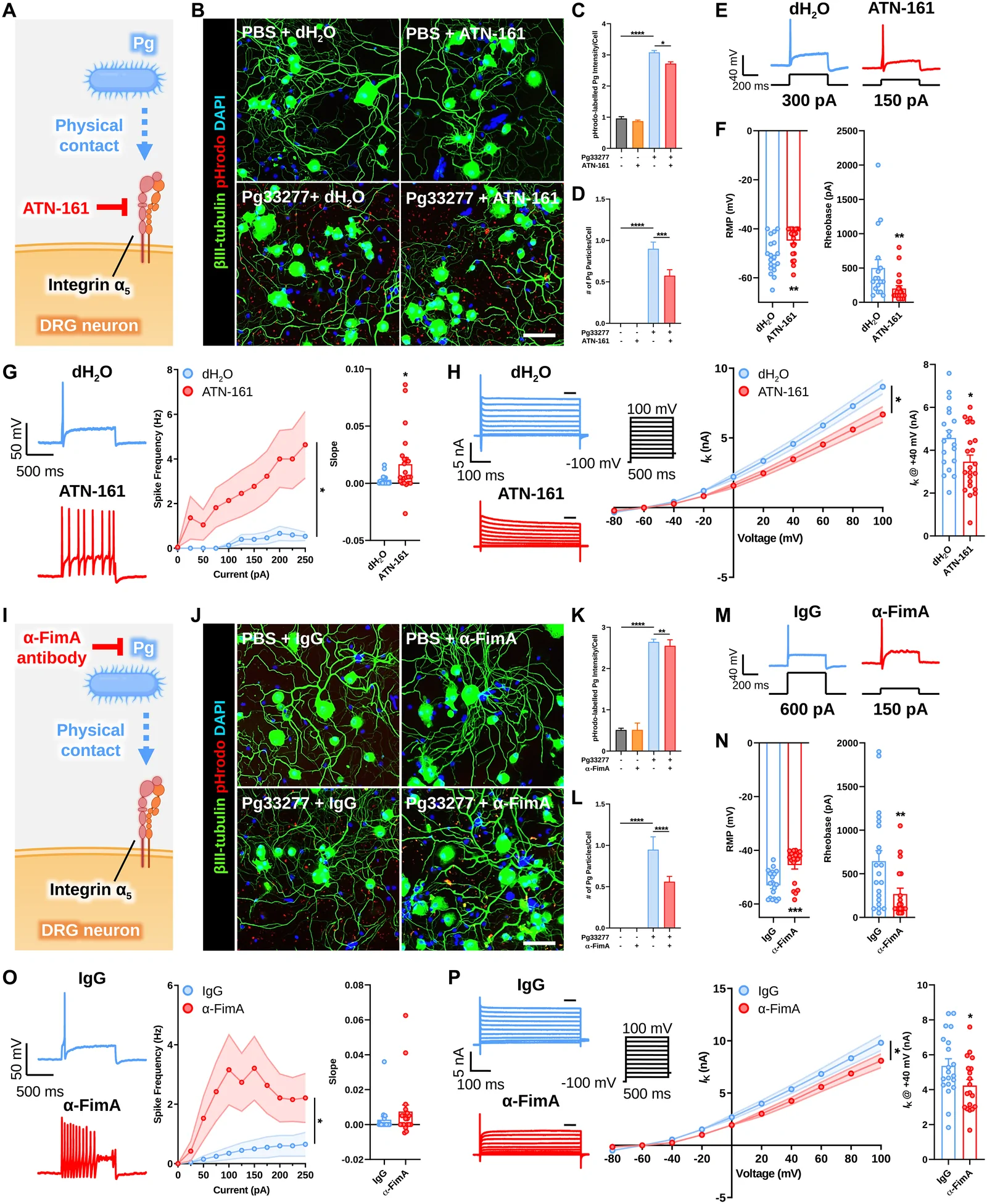
Integrin α_5_-FimA interaction mediates neuron-Pg contact to induce neuronal hypoexcitability. (**A** and **I**) Schematics of contact blockade using ATN-161 (A; created in BioRender. Chung, S. https://BioRender.com/r8no9qw) or α-FimA antibody (I; created in BioRender. Chung, S. https://BioRender.com/javqtkz). (**B** and **J**) Representative pHrodo-Pg33277 images with βIII-tubulin-/DAPI-stained DRG neurons after 6-hour co-culture ± ATN-161 or IgG/α-FimA. Scale bar: 50 μm. (**C** and **D**) pHrodo intensity and particle number/βIII-tubulin^+^ DAPI^+^ cell after ATN-161 blockade (*n* = 310/389/388/362 for PBS + dH_2_O/PBS + ATN-161/Pg33277 + dH_2_O/Pg33277 + ATN-161; C, *P* = 0.0381 & *P* < 0.0001; D, *P* = 0.0002 & *P* < 0.0001). (**E** to **H**) Rheobase-evoked action potentials (E), RMP/rheobase (F; *n* = 19/17 vs. 24/22; *P* = 0.0076/0.0027), spike frequency/slope (G; *n* = 15 vs. 22; *P* = 0.0436/0.0261), and *I*_K_ (H; *n* = 19 vs. 22; *P* = 0.0171 for *I*-*V* curve, *P* = 0.0299 at +40 mV) after ATN-161 blockade. (**K** and **L**) pHrodo intensity and particle number after α-FimA blockade (*n* = 417/466/429/448 for PBS + IgG/PBS + α-FimA/Pg33277 + IgG/Pg33277 + α-FimA; K, *P* = 0.0044 & *P* < 0.0001; L, *P* < 0.0001). (**M** to **P**) Rheobase-evoked action potentials (M), RMP/rheobase (N; *n* = 21 vs. 19; *P* = 0.0003/0.0075), spike frequency/slope (O; *n* = 20 vs. 19; *P* = 0.0227), *I*_K_ (P; *n* = 20 vs. 19; *P* = 0.0483 for *I*-*V* curve, *P* = 0.0436 at +40 mV) after α-FimA blockade. Imaging: two random images/group from two independent experiments; electrophysiology: three to four independent experiments. Kruskal-Wallis/Dunn’s tests in (C), (D), (K), and (L). Mann-Whitney tests in (F), (G, slope), (H, *I*_K_ at +40 mV), (N), (O, slope), and (P, *I*_K_ at +40 mV). Two-way ANOVA/Bonferroni’s tests in (G), (H), (O), and (P). Only significant comparisons are indicated. *P < 0.05, **P < 0.01, ***P < 0.001, ****P < 0.0001. Data are presented as mean ± S.E.M.

Having established that Pg-induced neuronal suppression requires direct contact, we next asked how this physical interaction is formed. Because Pg fimbriae have been reported to engage host α_5_β_1_ integrin during adhesion and invasion (*35*, *36*), and DRG sensory neurons express β_1_-class integrins including α_5_β_1_ (*37*, *38*), we tested whether Pg-DRG neuron contact depends on host integrin α_5_ by applying its antagonist ATN-161 during co-culture with pHrodo-labeled Pg33277 (Fig. 5A). ATN-161 markedly reduced Pg association with DRG neurons as shown by decreased pHrodo signal intensity and reduced numbers of pHrodo-positive particles associated with βIII-tubulin^+^ DAPI^+^ neurons (Fig. 5, B to D). These data indicate that integrin α_5_ contributes to the physical association between Pg and sensory neurons.

We next examined whether disruption of this contact was sufficient to restore neuronal function. Whole-cell patch-clamp recordings after 6 hours of Pg33277 co-culture showed that ATN-161 attenuated Pg-induced membrane hyperpolarization and reduced the increase in rheobase (Fig. 5, E and F). ATN-161 also restored AP firing in response to depolarizing current injection and normalized the slope of the frequency-current relationship (Fig. 5G). In voltage-clamp recordings, ATN-161 reduced the Pg-induced enhancement of outward *I*_K_, including *I*_K_ measured at +40 mV (Fig. 5H). Thus, blocking host-side integrin α_5_ engagement reduces Pg-neuron contact and functionally rescues Pg-induced neuronal hypoexcitability.

To independently test the bacterial-side component of this interaction, we next neutralized FimA using α-FimA antibody during Pg33277 co-culture (Fig. 5I). Similar to ATN-161, α-FimA antibody reduced pHrodo-labeled Pg association with DRG neurons, decreasing both pHrodo intensity and the number of pHrodo^+^ particles associated with βIII-tubulin^+^ DAPI^+^ neurons (Fig. 5, J to L). Functionally, α-FimA antibody reversed Pg-induced membrane hyperpolarization, rheobase elevation (Fig. 5, M and N), firing suppression (Fig. 5O), and *I*_K_ enhancement (Fig. 5P).

Together, these results demonstrate that Pg-induced neuronal suppression is not mediated by bacterial proximity alone, but requires an adhesion-dependent contact interface involving host integrin α_5_ and bacterial FimA. Disrupting either side of this interface reduces Pg attachment to DRG neurons and restores membrane excitability, supporting a model in which integrin α_5_-FimA-dependent contact is a critical upstream step in Pg-induced nociceptor silencing.

### Pg disrupts vesicle exocytosis machinery and Na^+^/K^+^-ATPase regulation in sensory neurons

**Fig. 6.**
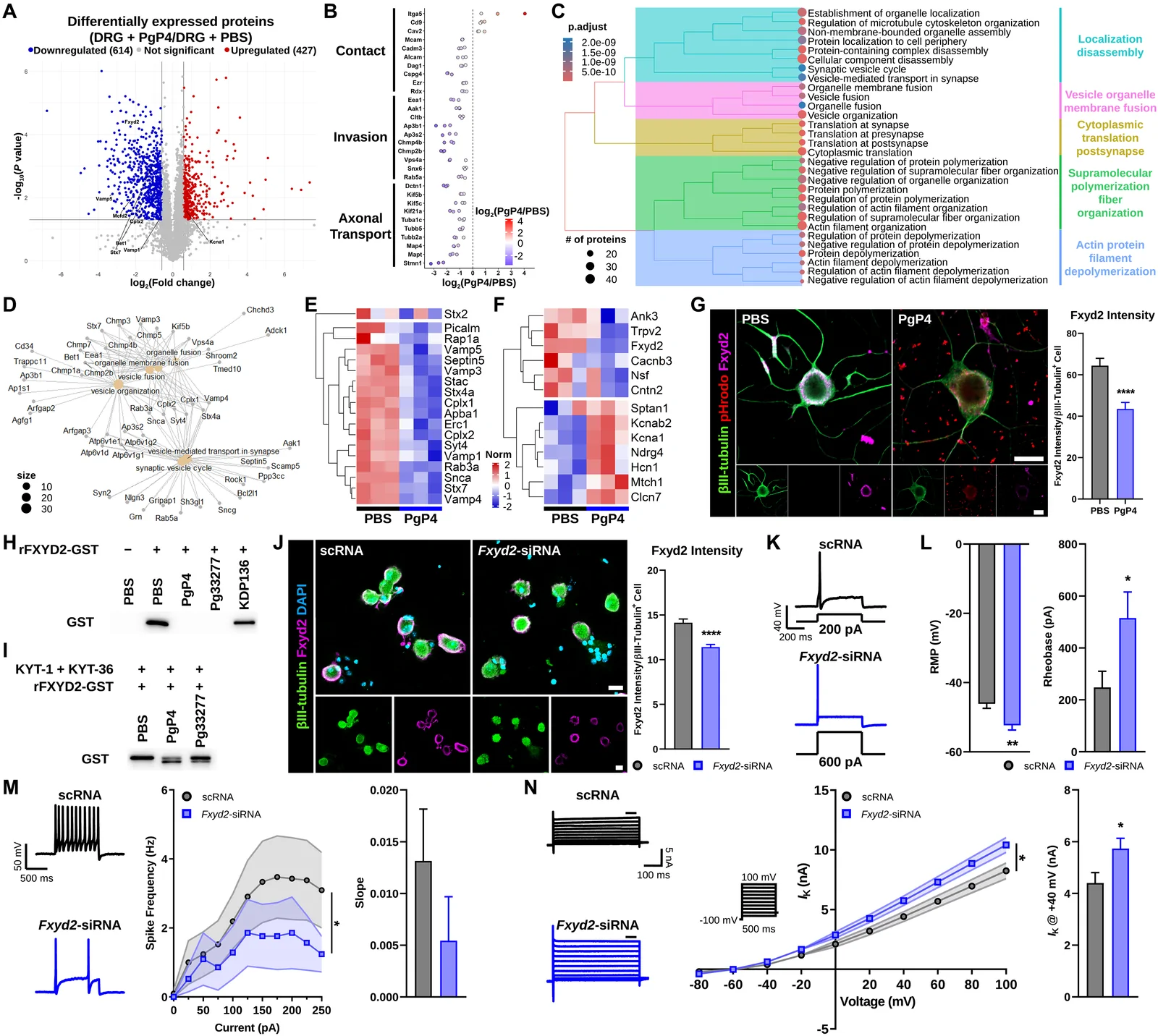
Vesicle exocytosis machinery and Fxyd2 are downregulated by Pg. (**A**) Volcano plot of differentially expressed proteins in PBS- and PgP4-co-cultured DRG neurons (|FC| ≥ 1.5, *P* < 0.05). (**B**) Replicate-level log_2_(PgP4/PBS) ratios for proteins grouped by contact-, invasion-related trafficking-, and axonal transport-associated categories. (**C**) GO tree plot of downregulated proteins. (**D**) Cnet plot of vesicle transport/fusion-related GO terms. (**E** and **F**) Heatmaps of SNARE/vesicle release proteins (E) and ion channels/pumps (F). Proteomics used three biological replicates per group. (**G**) Representative images and quantification of Fxyd2 intensity in PBS- or PgP4-treated DRG neurons (*n* = 36 for PBS & *n* = 29 for PgP4) from three independent experiments (*P* < 0.0001). Scale bars: 20 μm. (**H** and **I**) Western blot analysis of GST-tagged recombinant human FXYD2 (rFXYD2; 34.4 kDa) after 2-hour incubation with Pg strains (H) or Pg strains with KYT-1/KYT-36 (I), using an α-GST antibody. (**J**) *Fxyd2* knockdown validation in DRG neurons transfected with scRNA (*n* = 353) or *Fxyd2*-siRNA (*n* = 669) from two independent experiments (*P* < 0.0001). Scale bars: 20 μm. (**K** to **N**) Representative traces and quantification of rheobase-evoked action potentials (K), RMP/rheobase (L; *n* = 23 for scRNA & *n* = 27/26 for *Fxyd2*-siRNA; *P* = 0.0023/0.0216), spike frequency/slope (M; *n* = 21/group; *P* = 0.0416 for frequency), and *I*_K_ (N; *n* = 20 for scRNA & *n* = 26 for *Fxyd2*-siRNA; *P* = 0.0248 for *I*-*V* curve, *P* = 0.0183 at +40 mV) from two independent experiments. Mann-Whitney tests in (G), (J), (L), (M, slope), and (N, *I*_K_ at +40 mV). Two-way ANOVA with Bonferroni’s multiple comparisons test in (M, spike frequency) and (N, *I*_K_). Only significant comparisons are indicated. *P < 0.05, **P < 0.01, ****P < 0.0001. Data are presented as mean ± S.E.M.

To elucidate the molecular basis underlying the electrophysiological and functional changes induced by Pg, we performed label-free liquid chromatography-tandem mass spectrometry (LC-MS/MS)-based proteomic profiling of DRG neurons after 6 hours of PgP4 co-culture. Because this proteomic analysis was performed after 6 hours of Pg co-culture, these changes may include both early downstream responses to Pg-neuron engagement and secondary consequences of altered neuronal excitability, rather than representing direct primary effects of Pg. Among 4457 quantified proteins, 614 were significantly downregulated compared with PBS-treated controls (*P* < 0.05, fold change >1.5) (Fig. 6A). To better visualize the directionality and biological distribution of these changes, we further examined protein changes across individual biological replicates and grouped selected proteins into functional categories related to cell contact, invasion-associated trafficking, and axonal transport (Fig. 6B). This analysis showed that a subset of upregulated proteins was biased toward contact-related processes. Among these proteins, integrin α_5_, encoded by *Itga5*, showed the strongest increase in abundance within the contact-related category, consistent with the contact-dependent interaction between Pg and DRG neurons. However, these upregulated proteins did not form strongly coherent enriched pathways in the unbiased gene ontology (GO) analysis.

In contrast, downregulated proteins showed robust and functionally coherent enrichment in pathways related to neuronal vesicle trafficking and exocytosis. GO enrichment analysis revealed overrepresentation of terms associated with “synaptic vesicle cycle,” “vesicle-mediated transport in synapse,” “organelle membrane fusion,” “vesicle fusion,” “organelle fusion,” and “vesicle organization” (Fig. 6, C and D). Hierarchical clustering of vesicle-associated proteins specifically highlighted reduced abundance of key components of the exocytotic machinery, including members of the SNARE complex [syntaxins, complexins, and vesicle-associated membrane proteins (VAMPs)] in PgP4-treated neurons relative to controls (Fig. 6E). These data suggest that PgP4 disrupts the molecular machinery essential for synaptic vesicle trafficking and exocytosis, thereby impairing the release of neuropeptides such as CGRP from DRG neurons.

In addition to vesicle trafficking proteins, proteomic analysis also identified significant downregulation of Fxyd2, a γ subunit regulatory component of the Na^+^/K^+^-ATPase complex (Fig. 6, A and F). Decreased Fxyd2 expression was independently validated by ICC (Fig. 6G). To determine whether Fxyd2 could be targeted by live Pg, we incubated GST-tagged recombinant human FXYD2 (rFXYD2) with Pg strains for 2 hours and analyzed rFXYD2 integrity by Western blotting using an α-GST antibody. Live PgP4 and Pg33277 reduced the GST-tagged rFXYD2 signal, whereas the gingipain-null mutant KDP136 showed reduced proteolytic activity toward rFXYD2 (Fig. 6H). Furthermore, gingipain-specific inhibitors KYT-1 and KYT-36 attenuated the Pg-induced reduction of rFXYD2 signal (Fig. 6I), supporting gingipain-dependent proteolytic targeting of FXYD2.

To determine whether reduced Fxyd2 expression is sufficient to reproduce the electrophysiological phenotype induced by Pg, we performed siRNA-mediated knockdown of Fxyd2 in primary DRG neurons. Knockdown efficiency was validated by immunocytochemistry, which showed a significant reduction in Fxyd2 immunoreactivity in *Fxyd2*-siRNA-transfected neurons compared with scrambled RNA (scRNA) controls (Fig. 6J). Whole-cell patch clamp recordings after *Fxyd2* knockdown showed membrane hyperpolarization, increased rheobase, decreased spike frequency, and enhanced *I*_K_ compared with scRNA controls (Fig. 6, K to N), thereby recapitulating the electrophysiological phenotypes observed in Pg-treated neurons.

Fxyd2 is a known modulator of the Na^+^/K^+^-ATPase activity that maintains ionic gradients and contributes to the RMP (*39*). Its downregulation is therefore expected to enhance pump activity, leading to greater hyperpolarization and increased K^+^ driving force (*40*, *41*). Together with the observed suppression of vesicle exocytosis machinery, these findings support a model in which Pg-neuron contact induces gingipain-dependent targeting of Fxyd2 and downstream disruption of neuronal output pathways, thereby contributing to contact-dependent nociceptor silencing.

## DISCUSSION

**Fig. 7.**
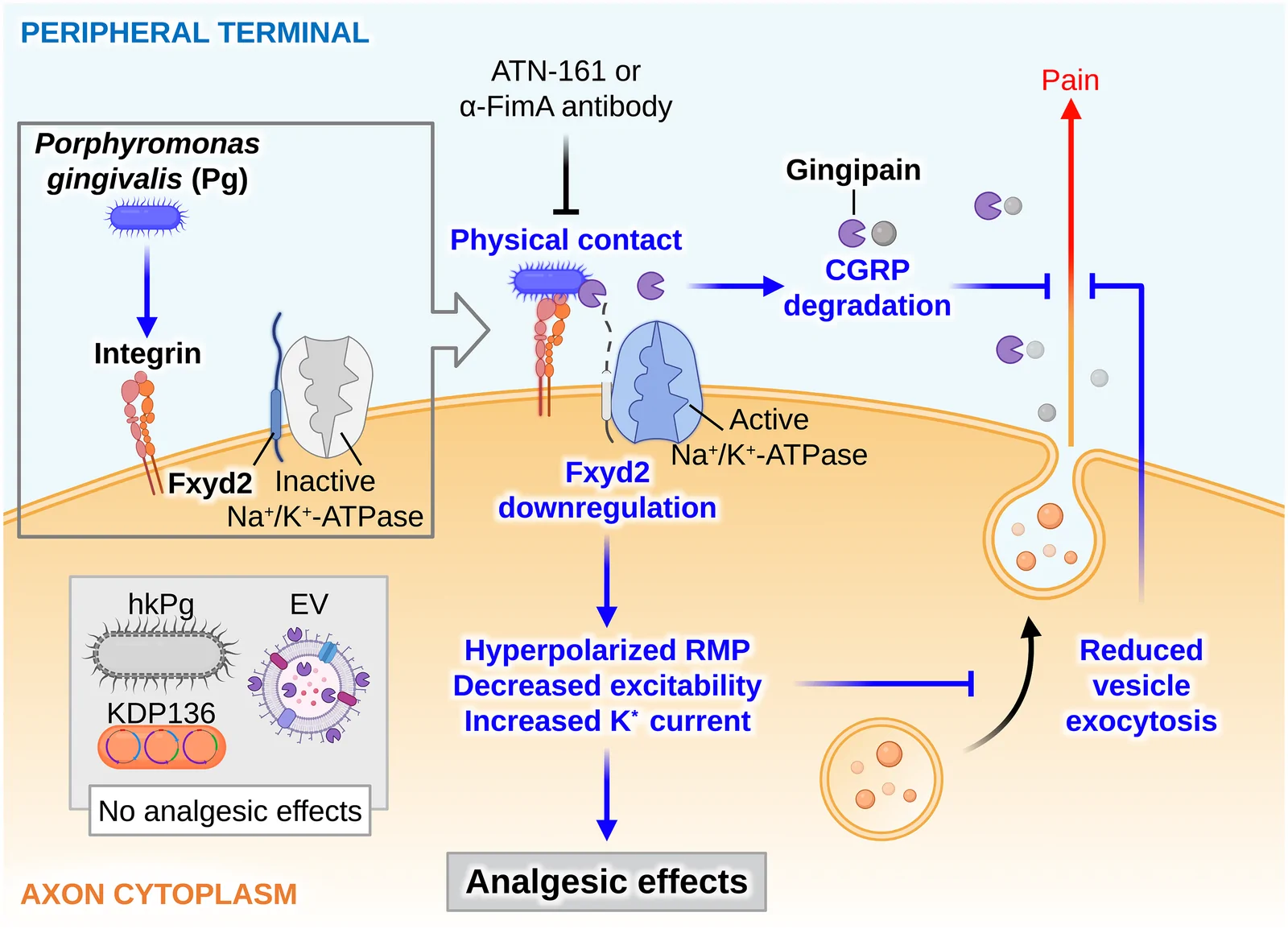
Graphical summary of integrin α_5_-FimA-dependent and gingipain-mediated suppression of nociceptor output by Pg. Pg establishes direct contact with DRG neurons through an integrin α_5_-FimA-dependent interface. This contact enables localized gingipain-dependent targeting and reduction of Fxyd2, a regulatory subunit of Na^+^/K^+^-ATPase, leading to membrane hyperpolarization, reduced excitability, increased K^+^ current, impaired vesicle exocytosis, reduced CGRP release, and suppression of inflammatory pain signaling (created in BioRender. Chung, S. https://BioRender.com/zw32d07).

Through behavioral, electrophysiological, imaging, and proteomic analyses, we identify a contact-dependent mechanism by which Pg suppresses sensory neuron activity and inflammatory pain. These findings provide a mechanistic explanation for the paradoxical dissociation between tissue inflammation and overt pain in periodontitis. Rather than merely failing to activate nociceptors, Pg actively suppresses nociceptor output through a non-canonical host-pathogen interaction. This analgesic effect required live bacteria and gingipain activity, but was not reproduced by heat-killed bacteria, gingipain-null mutants, or Pg-derived EVs. We further show that Pg-neuron contact is mediated, at least in part, through a host integrin α_5_-bacterial FimA interface, and that disruption of either side of this contact reduces bacterial association with DRG neurons and restores neuronal excitability. Downstream of this contact, our data support gingipain-dependent reduction of Fxyd2, a regulatory subunit of Na^+^/K^+^-ATPase, accompanied by membrane hyperpolarization, reduced excitability, enhanced outward K^+^ current, impaired vesicle exocytosis machinery, and reduced CGRP release. Together, these findings define an integrin α_5_-FimA-dependent and gingipain-mediated pathway by which Pg suppresses peripheral nociceptor signaling during inflammation (Fig. 7).

A central finding of this study is that Pg-induced nociceptor suppression requires direct bacterial-neuronal contact rather than diffusible bacterial products alone. Several observations support this conclusion. Heat-killed Pg failed to reproduce the analgesic phenotype, Pg-derived EVs were insufficient to induce analgesia, and Pg exposure did not elicit acute neuronal activation, inflammatory sensitization, or cytotoxicity (Fig. 2 and fig. S2). In addition, confocal imaging demonstrated close apposition of Pg to DRG neuronal membranes (Fig. 3, A and B), whereas transwell separation abolished Pg-induced membrane hyperpolarization, excitability suppression, outward K^+^ current enhancement, and CGRP reduction (Fig. 4 and fig. S4). These results indicate that intact Pg must physically engage sensory neurons to efficiently suppress nociceptor function.

The identification of the integrin α_5_-FimA axis further defines the molecular basis of this physical interaction. Pg fimbriae are well-established mediators of host-cell adhesion, and our data indicate that blockade of host integrin α_5_ with ATN-161 or neutralization of bacterial FimA with α-FimA antibody reduces Pg association with DRG neurons (Fig. 5). Importantly, both interventions attenuated Pg-induced membrane hyperpolarization, rheobase elevation, firing suppression, and outward K^+^ current enhancement. These findings indicate that Pg-neuron contact is not simply a consequence of bacterial proximity, but represents an adhesion-dependent interaction that is functionally required for neuronal silencing. This mechanism differs from previously described bacterial analgesic pathways mediated by diffusible toxins, such as mycolactone from *M. ulcerans* (*7*, *8*) and anthrax edema toxin from *B. anthracis* (*9*), which act through defined host receptors to suppress nociceptive signaling.

Direct Pg-neuron contact induced coordinated changes in neuronal excitability and neuropeptide output. At the level of intrinsic membrane properties, Pg shifted the RMP toward hyperpolarization, increased rheobase, reduced AP firing, and enhanced outward K^+^ currents (Fig. 4). At the level of neuronal output, Pg reduced CGRP release from DRG neurons, and this effect was abolished under transwell conditions (Fig. 4 and fig. S4). Proteomic profiling provided additional support for this contact-dependent model. Although both upregulated and downregulated proteins were detected after Pg exposure, a subset of upregulated proteins was associated with contact-related processes (Fig. 6B), consistent with active bacterial-neuronal engagement. In contrast, downregulated proteins showed coherent enrichment for vesicle trafficking, synaptic vesicle cycle, vesicle fusion, and exocytosis-related pathways (Fig. 6, C to E). These molecular changes provide a plausible basis for reduced neuropeptide secretion and suggest that Pg suppresses nociceptor output through coordinated effects on membrane excitability and vesicle release machinery.

Among the downregulated proteins, Fxyd2 emerged as a candidate mediator linking Pg contact to neuronal hypoexcitability. Fxyd2 encodes a γ subunit regulator of the Na^+^/K^+^-ATPase, which contributes to ionic gradient maintenance and RMP. PgP4 co-culture reduced Fxyd2 abundance in the proteomic dataset, and this reduction was independently validated by immunocytochemistry (ICC) (Fig. 6, A, F, and G). Biochemical analysis further showed that live Pg reduced GST-tagged rFXYD2, and that this effect was attenuated by the gingipain-specific inhibitors KYT-1 and KYT-36 (Fig. 6, H and I), supporting gingipain-dependent proteolytic targeting of Fxyd2. In parallel, siRNA-mediated *Fxyd2* knockdown, validated by ICC (Fig. 6J), recapitulated the major electrophysiological effects of Pg, including membrane hyperpolarization, increased rheobase, decreased AP firing, and enhanced K^+^ currents (Fig. 6, K to N). These findings support Fxyd2 reduction as a key downstream event contributing to Pg-induced suppression of sensory neuron excitability.

Based on these data, we propose that integrin α_5_-FimA-dependent contact establishes a spatially restricted host-pathogen interface at the neuronal membrane, enabling localized gingipain activity toward membrane-associated targets such as Fxyd2. Reduced Fxyd2 levels may alter Na^+^/K^+^-ATPase regulation, thereby shifting the RMP toward hyperpolarization and decreasing nociceptor responsiveness. This interpretation is consistent with previous reports showing that Fxyd2 expression is upregulated in inflammatory and neuropathic pain models and that Fxyd2-deficient mice exhibit reduced pain hypersensitivity (*39*, *42*, *43*). The concomitant suppression of CGRP release provides a functional correlate of this electrophysiological silencing. Thus, Pg-induced analgesia appears to arise from coordinated effects on membrane excitability and vesicle-mediated neuropeptide output downstream of direct bacterial-neuronal contact.

Although our findings support integrin α_5_-FimA-dependent contact as a major mechanism underlying Pg-induced nociceptor suppression, they do not exclude potential roles of soluble or EV-associated bacterial factors in other contexts. Previous studies have shown that Pg gingipains can cleave or activate PAR2 and thereby induce calcium signaling or inflammatory responses in non-neuronal cells, including neutrophils and microglia (*44*, *45*). However, in our DRG neuron culture system, live Pg did not evoke acute Ca^2+^ responses or enhance capsaicin- or inflammatory mediator-induced Ca^2+^ responses, arguing against a dominant acute PAR2-mediated neuronal activation or sensitization mechanism under these conditions. Pg EVs contain gingipains and other virulence-associated molecules that may influence non-neuronal cell types or broader host-pathogen communication. For example, the lipoprotein PG1881, recently identified as abundant in Pg EVs, has been implicated in bacterial communication and immune modulation (*46*), and bacterial lipids and lipopeptides can activate nociceptive neurons in other contexts (*47*, *48*). However, in our experimental system, EV administration did not reproduce the analgesic phenotype of live Pg, and transwell separation abolished Pg-induced neuronal modulation. Thus, EV-associated or diffusible factors appear insufficient to drive sensory neuron silencing under these conditions, although their contributions to other aspects of Pg-host interaction warrant further investigation.

Several mechanistic questions remain. First, although rFXYD2 assays support gingipain-dependent proteolytic targeting, the precise cleavage site and the relative contributions of Rgp and Kgp remain to be determined. In addition, although siRNA-mediated *Fxyd2* knockdown phenocopied several electrophysiological effects of Pg, whether restoration of Fxyd2 expression can attenuate Pg-induced neuronal suppression remains an important question for future studies. Second, direct measurement of Na^+^/K^+^-ATPase activity in Pg-treated DRG neurons will be required to establish how Fxyd2 reduction alters pump function and ionic homeostasis. Third, Pg exposure reduced multiple proteins involved in vesicle trafficking and exocytosis, but whether these changes represent direct consequences of bacterial proteolytic activity or secondary adaptations to neuronal hypoexcitability remains unresolved. Fourth, the ion-channel populations responsible for the enhanced outward K^+^ current remain to be identified. Finally, although confocal imaging and functional blockade support close Pg-neuron interaction, ultrastructural studies will be required to define the precise membrane interface between Pg and sensory neurons. Addressing these questions through targeted biochemical and electrophysiological analyses will refine our understanding of bacterial-neuronal communication and its impact on nociceptive processing during inflammation.

In summary, this study identifies Pg as a contact-dependent suppressor of nociceptor activity. In contrast to bacterial analgesic mechanisms mediated primarily by diffusible toxins, Pg establishes an integrin α_5_-FimA-dependent contact interface with sensory neurons, enabling localized gingipain-dependent targeting of Fxyd2 and suppression of neuronal output. This pathway results in membrane hyperpolarization, reduced excitability, impaired vesicle exocytosis machinery, and decreased CGRP release, thereby suppressing inflammatory pain signaling. Clinically, these findings provide a mechanistic explanation for why patients with periodontitis may experience little or no pain despite ongoing tissue destruction, underscoring the need for early diagnosis and intervention even in the absence of pain symptoms. More broadly, our findings expand the conceptual framework of host-microbe interactions by demonstrating that bacterial contact can directly silence peripheral sensory pathways and modulate pain perception during inflammation.

## MATERIALS AND METHODS

### Study approval

All animal and bacterial experiments were conducted and approved according to the Institutional Animal Care and Use Committee (IACUC) and the Institutional Biosafety Committee (IBC) at Seoul National University (SNU-200721-5-4 & SNUIBC-R200715–1-3) and Seoul National University Hospital (22–0110-S1A0 & 2201–004-004). The import and the use of KDP136 were approved by the Korea Disease Control and Prevention Agency (22-IMO-008 & 22-RDM-032).

### Animals

Young 5- to 6-week-old male C57BL/6 mice were purchased from Doo Yeol Biotech, South Korea and maintained in a 12-hour light/12-hour dark cycle environment with a constant control of temperature (22 ± 1°C) and humidity (55%). Standard lab chow and water were available ad libitum. For injection and behavioral assays, 6- to 8-week-old male mice were used. For the primary culture of DRG neurons, 5- to 8-week-old mice were used. Handling and care of the animals were in accordance with the Guideline for Animal Experiments, 2000, edited by the Korean Academy of Medical Sciences, which is consistent with the National Institutes of Health (NIH) Guidelines for the Care and Use of Laboratory Animals, revised 1996.

### Bacterial strains and culture

KUMC-P4, a clinical isolate of *P. gingivalis* (PgP4), from a periodontitis patient (*20*), *F. nucleatum* subsp. *nucleatum* (Fn; ATCC #25586), *V. atypica* (Va; ATCC #17744), and *S. aureus* (Sa; ATCC #29213) were obtained from Seoul National University, Seoul, South Korea. The type strain *P. gingivalis* (Pg33277; ATCC #33277) was obtained from the ADA Forsyth Institute, MA, USA. The gingipain-null (*kgp*^−^ *rgpA*^−^ *rgpB*^−^) mutant of Pg33277 (KDP136) was obtained from Nagasaki University, Nagasaki, Japan, under a material transfer agreement. Fn, Pg33277, and PgP4 were anaerobically (10% CO_2_, 10% H_2_, and 80% N_2_) grown at 37°C in enriched brain heart infusion (BHI) broth (BD, NJ) supplemented with 5 μg/ml hemin (Sigma-Aldrich, MO) and 1 μg/ml vitamin K (Sigma-Aldrich). KDP136 was grown in the same manner with the addition of 5 μg/ml yeast extract, 1 μg/ml L-cysteine, 20 μg/ml chloramphenicol, 10 μg/ml erythromycin, and 0.7 μg/ml tetracycline. Va was anaerobically grown in 188 *Veillonella* medium. Sa was aerobically cultured at 37°C in BHI broth. Bacteria were grown to late mid-exponential phase, pelleted, and resuspended in saline or PBS for both behavioral assays and in vitro studies at 2.5 × 10^8^ colony-forming unit (CFU)/ml. For fluorescent labelling, bacteria were stained with 2 μM CFSE (Molecular Probes, OR) for 15 minutes at 37°C or 10 μg/ml pHrodo Red dye (Invitrogen #P36600) for 30 minutes at RT. Heat-killed Pg (hkPg) was prepared by adjusting the concentration of live bacteria to the working concentration and then heating at 65°C for 2 hours. Heat inactivation was confirmed by the lack of colony formation 3 days after plating. For infection, 20 μl of bacteria (5 × 10^6^ CFU) suspended in saline or PBS were injected into the plantar surface of a hind paw with a 0.3-cc insulin syringe.

### Extracellular vesicle (EV) purification

Pg’s EVs were prepared as previously reported (*49*) with minor adjustments. Briefly, Pg was cultured at late mid-log phase (24 hours), and culture supernatants were harvested by centrifugation (10,000g, 4°C, 30 minutes). The supernatants were filtered using a 0.22-μm pore size membrane filter, and crude EVs were isolated by ultracentrifugation (160,000g, 4°C, 2 hours) using a Type 45 Ti rotor (Beckman Coulter, Brea, CA). Then, the crude EVs were dissolved in 1 ml saline and resuspended with 2 ml 60% OptiPrep to make 40% OptiPrep. The crude EV in 3 ml 40% OptiPrep was overlaid with 5 ml 35% OptiPrep and 5 ml 10% OptiPrep and subjected to buoyant density gradient ultracentrifugation (160,000g, 4°C, 4 hours) using an SW 40 Ti rotor (Beckman Coulter). Each 1.3 ml fraction was obtained from the top of the gradient sample (fractions #1–10). Fractions 4 and 5 were pooled, and EVs in fractions 4 and 5 were harvested by ultracentrifugation (120,000g, 4°C, 2 hours) using an SW 40 Ti rotor. Purified EVs were dissolved in 500 μl saline. The absence of bacterial cells in the EV preparation was confirmed by transmission electron microscopy (TEM).

### Mouse model of inflammatory pain

To mimic the chronic inflammation environment of periodontitis, CFA (1 mg/ml, 20 μl) was intraplantarly (i.pl.) injected in the ipsilateral hind paw as previously reported (*50*) with minor adjustments. Briefly, a single i.pl. CFA injection was done 3 days prior to the bacterial infection or EV injection.

### Hargreaves’ test

Mice were habituated in the transparent acrylic cages for at least 2 hours/day for 3 consecutive days prior to behavioral assays. For thermal sensitivity, mice were placed on a glass plate of a Hargreaves’ apparatus (IITC Life Science) without a heated base for the CFA model & with a heated base for naïve mice. The withdrawal latency was measured by applying the heat source (active intensity of 35% for the CFA model & 25% for naïve mice) to the injected area with a cut-off time of 20 seconds.

### von Frey assay

For mechanical sensitivity, mice were placed on an elevated metal mesh floor, and the von Frey filaments (0.04–2.0 g; Stoelting) were applied to the injection area (3-, 24-, and 48-hour post-injection for the CFA model). The 50% withdrawal threshold was determined by the up-down method. Baseline (BL) assessment for all evoked pain tests was done after the habituation period and before the injection, and animals were randomly grouped so that group mean baseline and/or CFA thresholds were within similar error ranges. Behavioral scoring was done with the observer blind to the treatment groups.

### Spontaneous pain test

To assess spontaneous pain-like behavior following bacterial injection, mice were returned to the transparent acrylic cages after habituation and video-recorded for 60 minutes at 6 hours post-injection. The cumulative duration of licking and/or biting directed toward the injected site was quantified from the video recordings.

All behavioral assessments were conducted by an observer blind to the treatment groups. Hind paw thickness was measured with an electronic caliper at the end of the assays.

### H&E staining

The plantar surface of the ipsilateral hind paw skin was sampled with a 3-mm punch biopsy. After 15 minutes of 4% PFA (T&I Tech&Innovation, Gangwon-do, South Korea) post-fixation at room temperature (RT), the tissue samples were transferred into 30% sucrose (Sigma-Aldrich) for cryoprotection, embedded in OCT compound, cryosectioned at 10-μm thickness with Cryocut Microtome (Leica Microsystems, Bensheim, Germany), and then stained with hematoxylin and eosin (Abcam, Cambridge, United Kingdom) in the standard method. All sections were imaged by a bright-field light microscope (Leica), and the number of inflammatory cells per area was quantified using ImageJ (version 1.53 K, National Institutes of Health, USA).

### Immunofluorescence staining

Tissue sections at 20-μm thickness were washed in PBS and blocked with 4% normal horse serum (NHS, Vector Laboratories, Newark, CA) in 0.3% Triton X-100 (TX-100, Thermo Fisher Scientific) in PBS for 1 hour at RT in a humidified chamber. Slides were then incubated with rabbit α-βIII-tubulin antibody (1:500, Sigma-Aldrich) in the blocking solution for 2 days at RT and Cy3-conjugated donkey α-rabbit antibody (1:500, Jackson ImmunoResearch, West Grove, PA) in 0.3% TX-100 in PBS for 2 hours at RT. DAPI staining (1 μg/ml in dH_2_O, Sigma-Aldrich) was done for 10 minutes at RT before mounting a coverslip with mounting medium (Vector Laboratories). Fluorescence images were acquired on a laser-scanning confocal microscope (LSM 700, Zeiss, Jena, Germany) with Zen software (version 8.1, Zeiss) and imaged as a stack of 8 sequential z sections (20-μm depth). For the analysis of intraepidermal nerve fiber (IENF) density, βIII-tubulin-positive fibers that crossed the basement membrane were counted over the length of the stratum corneum as described (*51*) in the maximum intensity projection images. Randomized 4 images were analyzed per mouse.

### Primary culture of DRG neurons

Primary culture of DRG neurons was performed as previously reported (*52*) with minor adjustments. Briefly, after the mouse was euthanized with isoflurane, DRG were acutely dissected on ice-cold Hank’s balanced salt solution (HBSS) with 20 mM HEPES (Gibco; Thermo Fisher Scientific, Waltham, MA). Then, ganglia were digested for 40 minutes in collagenase A (1 mg/ml, Roche, Risch-Rotkreuz, Switzerland) and dispase II (2.4 U/ml, Roche) mixture in HBSS-HEPES-buffered solution at a 37°C incubator with 5% CO_2_. Then, 0.25% trypsin (Gibco) in HBSS was applied for 7 minutes. DRG were mechanically dissociated with trituration by fire-polished Pasteur pipettes in Dulbecco’s modified Eagle’s medium (DMEM) containing DNase I (Sigma-Aldrich). The cell suspension was then carefully layered over 15% bovine serum albumin (BSA) in Ham’s F-12 (Welgene, South Korea) to isolate neurons. Cells were resuspended with neurobasal medium (Gibco) containing B-27 supplement (Gibco), L-glutamate (Sigma-Aldrich), and penicillin-streptomycin (Gibco). 3000 DRG neurons were plated on 10 mm-diameter coverslips coated with poly-D-lysine (100 μg/ml, Sigma-Aldrich) for both calcium imaging and whole-cell patch clamp recording. For immunocytochemistry (ICC), 2000 DRG neurons were plated on 10 mm-diameter coverslips or confocal dishes coated with poly-D-lysine (10 μg/ml) and laminin (10 μg/ml, Sigma-Aldrich). For CGRP ELISA, 1 to 4 × 10^4^ DRG neurons/well were plated in a 24-well plate or in the bottom wells of a transwell plate coated with poly-D-lysine (10 μg/ml) and laminin (10 μg/ml).

### DRG & bacteria co-culture and contact blockade assay

Cultured DRG neurons were washed once with neurobasal medium, and 200 μl of bacteria (5 × 10^6^ CFU for ICC and 5 × 10^7^ CFU for calcium imaging, whole-cell patch clamp recording, ELISA, and contact blockade assay) were added directly over DRG neurons or in the upper well of a transwell plate for the indicated hours of co-culture at a 37°C incubator with 5% CO_2_. For the contact blockade assay, 10 μM ATN-161 (Tocris Bioscience, Bristol, United Kingdom) was pre-treated to DRG neurons for 30 minutes at a 37°C incubator with 5% CO_2_, or 10 μg/ml α-FimA antibody (Absolute Antibody, Redcar, United Kingdom) or its isotype control was pre-treated to Pg strains for 30 minutes at RT prior to indicated co-culture hours.

### *Fxyd2*-siRNA reverse transfection

For *Fxyd2* knockdown, primary DRG neurons were reverse transfected with *Fxyd2*-targeting siRNA or scrambled RNA (scRNA) for 24 hours prior to whole-cell patch-clamp recordings. 5 pmol *Fxyd2*-siRNA or scRNA (Bioneer, South Korea) in Opti-MEM (Gibco) was combined with RNAiMAX (Invitrogen) in Opti-MEM to generate transfection complex and incubated for 10 minutes at RT. For reverse transfection, siRNA transfection complex was added directly onto each coverslip immediately before plating 2000 dissociated DRG neurons. After cell attachment, additional complete Neurobasal culture medium without the antibiotics was added. Knockdown efficiency was validated by Fxyd2 immunocytochemistry.

### Ratiometric calcium imaging

Cultured DRG neurons were used for calcium imaging after incubation overnight. Cells were loaded with 2 μM Fura-2 a.m. (Thermo Fisher Scientific) in DMEM (Gibco) for 40 minutes at a 37°C incubator with 5% CO_2_. Images were acquired through a DL-604 M-VP CCD camera (Andor Technology, Belfast, United Kingdom) coupled to an Eclipse TE-2000 inverted microscope (Nikon, Tokyo, Japan) while a Lambda DG-4 plus ultraviolet light source (Sutter Instruments, Novato, CA) was used to excite Fura-2 by alternating 340 nm and 380 nm wavelength lights. Mean fluorescence intensity ratios (F340/F380) were measured with MetaFluor software (version 7.8.13.0, Molecular Devices, San Jose, CA). Bath solution (2 mM Ca^2+^-HBSS containing [in mM] 140 NaCl, 5 KCl, 2 CaCl_2_, 1 MgCl_2_, 10 HEPES, and 10 _D_-glucose) was adjusted to pH 7.4 with 5 N NaOH, osmolarity 300 ± 5 mOsm. In all experiments, an increase in F340/F380 at a minimum of ≥20% of the maximal amplitude (in 50 mM KCl) and/or 0.2 absolute value change was considered as a positive response to agonists.

### Optical membrane potential monitoring with FluoVolt

Cultured DRG neurons were used for live optical monitoring of membrane potential after incubation overnight. Cells were loaded with 1X FluoVolt dye (Invitrogen) in the bath solution for 30 minutes at RT in the dark according to the manufacturer’s instructions. Images were acquired through a laser scanning confocal microscope (LSM980, Carl Zeiss, Germany) every 10 seconds for 2 hours with controlled humidity and CO_2_ concentration (5%) in a live cell imaging chamber, and 50 mM KCl was added at the end for positive neuronal control.

### Whole-cell patch clamp recording

Overnight-cultured DRG neurons co-cultured with PgP4 (CFSE-labelled for transwell culture only) for 6 hours were examined under a 40X microscope (BX51, Olympus, Tokyo, Japan) equipped with infrared differential interference contrast (IR-DIC). Fluorescent signals from CFSE-positive neurons were detected using GFP wavelengths. Patch pipettes (tip resistance: 4–6 MΩ) were pulled from borosilicate capillaries (BF150–86-10, Sutter Instruments, Novato, CA, USA) using a P-97 puller (Sutter Instruments). Pipettes were filled with an internal solution containing the following (in mM): 123 K^+^-gluconate, 18 KCl, 10 NaCl, 3 MgCl_2_, 2 Na_2_ATP, 0.3 GTP-Na, and 0.2 EGTA, adjusted to pH 7.4 with KOH. The external solution for current-clamp experiments contained (in mM): 140 NaCl, 5 KCl, 2 CaCl_2_, 1 MgCl_2_, 10 HEPES, and 10 _D_-glucose, adjusted to pH 7.4 and with an osmolarity of 300 mOsm. For current-clamp recordings, neuronal excitability was assessed using two distinct step current injection protocols. To determine action potential (AP) threshold and rheobase, step current injections were delivered in 50-pA increments (500 ms duration) until the first AP was elicited. In neurons exhibiting higher rheobase, particularly following Pg co-culture, current steps were increased in 100-pA increments up to a maximum of 1000 pA to reliably identify spike initiation. Spike frequency was measured by applying step current injections from 0 to 250 pA in 25-pA increments (1 s duration). The number of APs evoked at each current step was quantified to generate frequency-current relationships. For voltage-clamp recordings of the K^+^ current-voltage relationship, 500-ms voltage steps from −80 mV to +100 mV were applied in 20-mV increments at 200-ms intervals. All recordings were performed at RT. Data acquisition was performed using pClamp 11 software (Molecular Devices) and a Multiclamp 700B amplifier (Molecular Devices). Signals were low-pass filtered at 10 kHz and digitized at 20–100 kHz using a Digidata 1550B digitizer (Molecular Devices). Neurons were excluded from analysis if access resistance (R_a_) exceeded 30 MΩ or RMP was less negative than −40 mV. R_a_ was not compensated during current-clamp experiments, and no liquid junction potential correction was applied.

### Immunocytochemistry (ICC)

Cells were fixed with 4% PFA for 15 min at 4°C and then permeabilized with 0.1% TX-100 in PBS for 15 min at 4°C. Cells were blocked with 4% NHS in 0.1% TX-100 in PBS for 1 hour at RT and incubated with rabbit α-βIII-tubulin antibody (1:2000, Sigma-Aldrich) and/or mouse α-Fxyd2 antibody (1:2000, Abnova, Taipei City, Taiwan) in the blocking solution overnight at 4°C. Incubation with Alexa Fluor 488-conjugated donkey α-rabbit antibody (1:2000, Jackson ImmunoResearch, West Grove, PA) and/or Alexa Fluor 647-conjugated donkey α-mouse antibody (1:2000, Jackson ImmunoResearch) in 0.1% TX-100 in PBS was done for 2 hours at RT. Fluorescence images were acquired on a laser-scanning confocal microscope (LSM 980, Zeiss) with Zen software (Zeiss) to visualize contact and infiltration of pHrodo in βIII-tubulin-/DAPI-positive DRG neurons and reconstructed in 3D. For contact blockade assay, fluorescence images were acquired on a digital inverted fluorescence microscope (DMi8, Leica) with LAS X software (Leica) to visualize contact of pHrodo in βIII-tubulin-/DAPI-positive DRG neurons.

### TUNEL assay

Cell death in DRG neurons was detected by using the TUNEL assay kit (C10618, Invitrogen) according to the manufacturer’s instructions after co-culturing with PgP4 or Pg33277 for 6 hours. Standard ICC procedure was followed.

### CGRP enzyme-linked immunosorbent assay (ELISA)

The culture medium was renewed with a fresh neurobasal medium immediately before bacterial co-culture or ligand treatment. The culture supernatant was collected 6 hours after the co-culture. The culture supernatant was centrifuged at 13,000 rpm for 1 min at 4°C, and 500 μl supernatant was collected to be kept at −80°C until use. The concentration of calcitonin gene-related peptide (CGRP) in the cultured DRG neurons was measured by using a commercial CGRP ELISA kit (Bertin Bioreagent, Montigny-le-Bretonneux, France) according to the manufacturer’s instructions. Samples were loaded in duplicates, and the limit of detection for CGRP was 0.7 pg/ml.

### IL-1β ELISA

IL-1β levels in the hind paw skin 2-day post-injection samples was measured using an ELISA kit (R&D Systems, MN, USA) according to the manufacturer’s instructions.

### Transmission electron microscopy (TEM)

For fimbriae visualization, bacterial cells in suspension were fixed with 2.5% glutaraldehyde. 20 μl of fixed cells was dropped onto 150-mesh copper grids (G-150 Cu, EMS) and incubated for 1 min. After negative staining with 2% uranyl acetate, the samples were imaged with JEM-1400 Flash transmission electron microscope (JEOL, Japan) at 120 kV.

### Recombinant human FXYD2 (rFXYD2) proteolysis assay

To examine whether FXYD2 can be directly targeted by Pg protease activity, GST-tagged recombinant human FXYD2 (rFXYD2-GST; Creative Biomart, Shirley, NY) was incubated with live Pg strains. Briefly, 1 μg rFXYD2-GST was mixed with PBS, PgP4, Pg33277, or KDP136 (5 × 10^7^ CFU) bacterial suspensions and incubated for 2 hours at RT. For gingipain inhibition experiments, Pg strains were pre-treated with the gingipain-specific inhibitors KYT-1 and KYT-36 (10 μM each, Peptide Institute, Osaka, Japan) for 30 minutes at RT before incubation with rFXYD2-GST. After incubation, samples were mixed with Laemmli sample buffer, boiled, separated by SDS-PAGE, and transferred to PVDF membranes. GST-tagged rFXYD2 was detected using a mouse α-GST antibody (1:1000, Invitrogen) in 2% skim milk in 0.1% TBST at 4°C overnight after 1 hour blocking in 2% skim milk in 0.1% TBST at RT. Protein bands were visualized using enhanced chemiluminescence after 2-hour incubation with horse α-mouse HRP antibody (1:500, Vector Laboratories) in 0.1% TBST at RT. Reduction of the GST-reactive rFXYD2 band was interpreted as proteolytic loss of rFXYD2 signal.

### RNA isolation, cDNA synthesis, and quantitative reverse transcription polymerase chain reaction (qRT-PCR)

RNA was isolated with 800 μl TRIzol (Invitrogen) from DRG lysates after 6-hour co-culture with PgP4. After adding 200 μl chloroform, the samples were centrifuged at 13,000 rpm at 4°C for 15 min. The top aqueous phase was transferred to 500 μl ice-cold isopropanol-containing tubes and incubated at RT for 10 min. Then, the tubes were centrifuged at 13,000 rpm at 4°C for 15 min. The pellet was washed twice with 1 ml 70% ethanol and then air-dried before resuspending with 20 μl DepC-treated dH_2_O. The quantity and quality of the sample were evaluated using a Nanodrop 2000 spectrophotometer (Thermo Fisher Scientific, Waltham, MA, USA). The RNA was reverse-transcribed to cDNA using M-MLV RT (28025013, Invitrogen) according to the manufacturer’s instructions. 84 genes involved in various signalling pathways were screened using a qPCR screening kit (SM-0147, Bioneer) with SYBR green PCR master mix (4367659, Thermo Fisher Scientific) and StepOnePlus real-time PCR system (Thermo Fisher Scientific). Experiments were performed according to the manufacturer’s instructions. The relative expression of screened genes was calculated using the 2^-ΔΔCt^ method.

### Protein preparation and digestion for proteomics

For proteomic analysis, DRG neurons were isolated from 4 mice per preparation and pooled prior to culture. Cells were then divided into PBS and 1.25 × 10^8^ CFU PgP4 treatment groups and cultured under identical conditions. This procedure was repeated on independent experimental days. For each biological replicate, lysates from two independent preparations were combined at the protein quantification stage. Using this approach, 3 independent biological replicates (*n* = 3/group; total 24 mice) were analyzed by mass spectrometry. Protein sample concentration was quantified using the Pierce BCA protein assay kit (Thermo Fisher Scientific). The digestion step was performed using filter-aided sample preparation (FASP) on a Microcon 30 K centrifugal filter device (Millipore, Billerica, MA, USA). Each sample was reduced by incubating with tris(2-carboxyethyl)phosphine (TCEP) at 37°C for 30 min and alkylated with iodoacetic acid (IAA) at 25°C for 1 hour in the dark. After washing with lysis buffer and 50 mM ammonium bicarbonate (ABC) sequentially, the proteins were digested with trypsin [enzyme to protein ratio of 1:50 (w/w)] at 37°C for 18 hours. The resulting peptide mixtures were collected into new tubes, and trypsin was inactivated by acidifying with 15 μl formic acid (Honeywell, Charlotte, NC, USA). The digested peptides were desalted using C_18_ spin columns (Harvard Apparatus, Holliston, MA, USA), and the peptides were eluted with 80% acetonitrile in 0.1% formic acid in water.

### LC-MS/MS analysis

LC-MS/MS analysis was performed using a previously described workflow with minor modifications (*53*). The prepared samples were resuspended in 0.1% formic acid in water and analyzed using a Q-Exactive Orbitrap hybrid mass spectrometer (Thermo Fisher Scientific, Waltham, MA, USA) along with an Ultimate 3000 system (Thermo Fisher Scientific, Waltham, MA, USA). We used a 2 cm × 75 μm ID trap column packed with 3 μm C_18_ resin and a 50 cm × 75 μm ID analytical column packed with 2 μm C_18_ resin to separate peptides depending on the peptides’ hydrophobicity. The mobile phase solvents consisted of (A) 0.1% formic acid in water and (B) 0.1% formic acid in 80% acetonitrile, and the flow rate was fixed at 300 nl/min. The gradient of mobile phase was as follows: 4% solvent B in 14 min, 4–15% solvent B in 61 min, 15–28% solvent B in 50 min, 28–40% solvent B in 20 min, 40–96% solvent B in 2 min, holding at 96% of solvent B in 13 min, 96–4% solvent B in 1 min, and 4% solvent B for 24 min. A data-dependent acquisition method was adopted, and the top 10 precursor peaks were selected and isolated for fragmentation. Ions were scanned in high resolution (70000 in MS1, 17500 in MS2 at *m/z* 400), and the MS scan range was 400–2000 *m/z* at both the MS1 and MS2 levels. Precursor ions were fragmented with 27% normalized collisional energy (NCE). Dynamic exclusion was set to 30 s.

### Proteomic data analysis

Proteomic data processing and downstream analysis were performed using a previously described workflow with minor modifications (*53*). Thermo MS/MS raw files of each analysis were searched by using Proteome Discoverer software (version 2.5, ThermoFisher), and the Mouse database was downloaded from Uniprot. The appropriate consensus workflow included a peptide-spectrum match (PSM) validation step and SEQUEST HT process for detection as a database search algorithm. The search parameters were set up as follows: 10 ppm of tolerances of precursor ion masses, 0.02 Da fragment ion mass, a maximum of two missed cleavages with trypsin enzyme. The dynamic modification on peptide sequence was as follows: static carbamidomethylation of cysteine (+57.012 Da), variable modifications of methionine oxidation (+15.995 Da), acetylation of protein N terminus (+42.011 Da), and carbamylation of protein in N terminus (+43.0006 Da). After searching, the data results below 1% of FDR were selected and filtered for at least 6 more peptides in length.

Raw proteomic data were processed and analyzed using R (version 4.4.1). Data preprocessing included normalization based on the total peptide amount method, where the sum of peptide group intensities was calculated for each sample, and the maximum sum across all files was used to compute the normalization factor. Log_2_ transformation was applied to stabilize variance, and statistical analysis was performed using *t*-tests to compare protein abundances between groups, applying a fold-change cutoff of 1.5 and a significance threshold of *P* < 0.05. Visualizations such as volcano plots and heatmaps were generated using ggplot2 and ComplexHeatmap packages, respectively. Functional annotation of downregulated proteins was carried out using gene ontology (GO) enrichment analysis with the enrichGO function in clusterProfiler, using a *P* value cutoff of 0.01 and a *q*-value cutoff of 0.05. The top 30 enriched GO terms were visualized through hierarchical tree plots, and cnet plots were generated to illustrate the relationships between key GO terms and their associated proteins.

### Statistical analysis

Statistical tests were selected based on data distribution, sample size, and experimental design. For time-course behavioral experiments, two-way ANOVA with Bonferroni’s post hoc test was used to assess treatment- and time-dependent effects. For two-group comparisons or endpoint analyses, the Mann-Whitney test was used when non-parametric analysis was appropriate. For comparisons among more than two groups, the Kruskal-Wallis test followed by Dunn’s multiple-comparisons test was used. The specific statistical test used for each experiment is indicated in the corresponding figure legend. All statistical analyses were performed using Prism software (version 8.4.3, GraphPad), except for the proteomic data analysis. Statistical significance was set at *P* < 0.05, and all data are presented as mean ± S.E.M.
