# Supplementary material for: Direct cellular contact by Porphyromonas gingivalis suppresses nociceptor excitability and induces analgesia during inflammation
Source: Sci Adv. 2026 Sep 25;12(39):eaee8088. doi: 10.1126/sciadv.aee8088 (PMC13614394; doi:10.1126/sciadv.aee8088)
Supplement: Supplementary file 1 [file sciadv.aee8088_sm.pdf]

Science Advances
AAAS

Supplementary Materials for

Direct cellular contact by *Porphyromonas gingivalis* suppresses nociceptor excitability and induces analgesia during inflammation

Sena Chung *et al.*

Corresponding author: Seog Bae Oh, odolbae@snu.ac.kr

Sci. Adv. **12**, eaee8088 (2026)
DOI: 10.1126/sciadv.aee8088

This PDF file includes:

Figs. S1 to S4

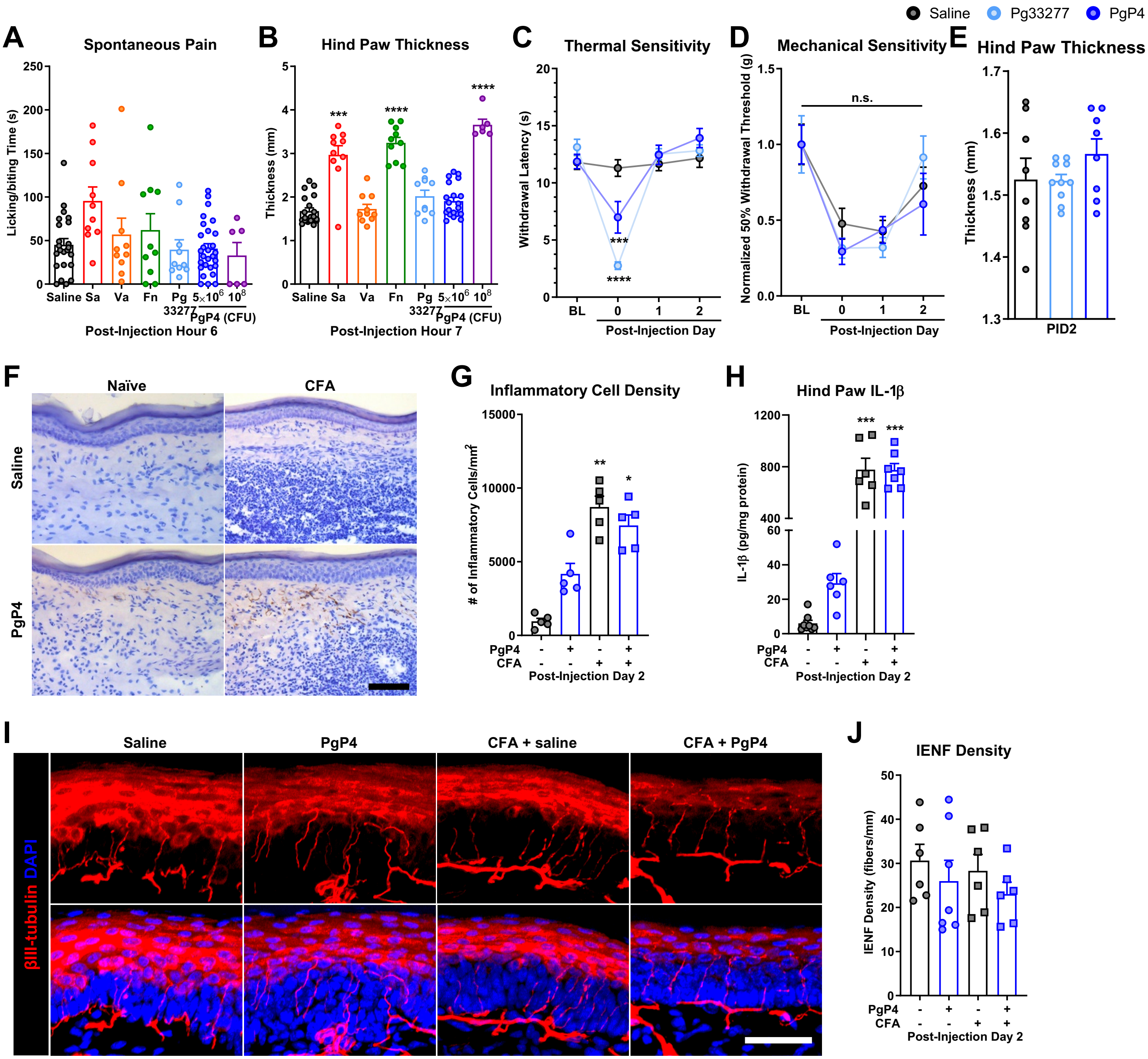

Fig. S1. Pg does not induce pain under naïve conditions and does not alter inflammation or nerve degeneration, related to Figure 1. (**A**) Spontaneous pain measured by quantifying licking/biting time of hind paw 6 hours after an intraplantar injection of saline (n = 23), Sa (n = 10), Va (n = 10), Fn (n = 10), Pg33277 (n = 10), 5 × 10^6 CFU PgP4 (n = 28), or 10^8 CFU PgP4 (n = 6). (**B**) Hind paw thickness measured by

caliper 7 hours after an intraplantar injection of saline (n = 20), Sa (n = 10), Va (n = 10), Fn (n = 10), Pg33277 (n = 10), 5 × 10^6 CFU PgP4 (n = 20), or 10^8 CFU PgP4 (n = 6) (P = 0.0007 for Sa, P < 0.0001 for Fn, and P < 0.0001 for 10^8 CFU PgP4 vs. saline). (**C**) Thermal sensitivity measured by Hargreaves' test at 0 (3 hours post-injection), 1, and 2 days after intraplantar injection of saline (n = 9), Pg33277 (n = 10), or PgP4 (n = 10) (P < 0.0001 for Pg33277 and P = 0.001 for PgP4 vs. saline at post-injection day (PID) 0). (**D**) Mechanical sensitivity measured by von Frey assay at 0 (3 hours post-injection), 1, and 2 days after intraplantar injection of saline (n = 14), Pg33277 (n = 10), or PgP4 (n = 13). No significant differences were observed between Pg33277 or PgP4 groups and saline at any post-injection time point. (**E**) Hind paw thickness measured by caliper at PID2 of saline (n = 8), Pg33277 (n = 10), or PgP4 (n = 8). (**F**) Representative H&E-stained images of naïve and CFA-preinjected hind paw skin sections at PID2. Scale bar: 100 μm. (**G**) Inflammatory cell density quantified by the number of inflammatory cells in dermis of naïve and CFA-preinjected hind paw skin section at PID2 of saline or PgP4 injection (n = 5/group) from two independent experiments (P = 0.0021 for CFA + saline and P = 0.0197 for CFA + PgP4 vs. saline). (**H**) IL-1β levels measured by ELISA in naïve and CFA-preinjected hind paw skins at PID2 of saline (n = 8), PgP4 (n = 6), CFA + saline (n = 6), and CFA + PgP4 (n = 7) (P = 0.0008 for CFA + saline and P = 0.0004 for CFA + PgP4 vs. saline). (**I**) Representative βIII-tubulin-stained images of naïve and CFA-preinjected hind paw skin sections at PID2 of saline or PgP4 from two independent experiments. Scale bar: 50 μm. (**J**) IENF density quantified by the number of βIII-tubulin-stained fibers crossing the basement membrane/length of the stratum corneum in saline (n = 6), PgP4 (n = 7), CFA + saline (n = 6), and CFA + PgP4 (n = 6). Kruskal-Wallis test with Dunn's

multiple comparisons test in (A), (B), (E), (G), (H), and (J). Two-way ANOVA with Bonferroni's multiple comparisons test in (C). Only significant comparisons are indicated. *$P < 0.05$, **$P < 0.01$, ***$P < 0.001$, ****$P < 0.0001$. Data are presented as mean ± S.E.M. CFA, complete Freund's adjuvant; Sa, *Staphylococcus aureus*; Va, *Veillonella atypica*; Fn, *Fusobacterium nucleatum*; Pg33277, *Porphyromonas gingivalis* ATCC #33277 strain; PgP4, *Porphyromonas gingivalis* KUMC-P4 strain (clinical isolate); n.s., not significant.

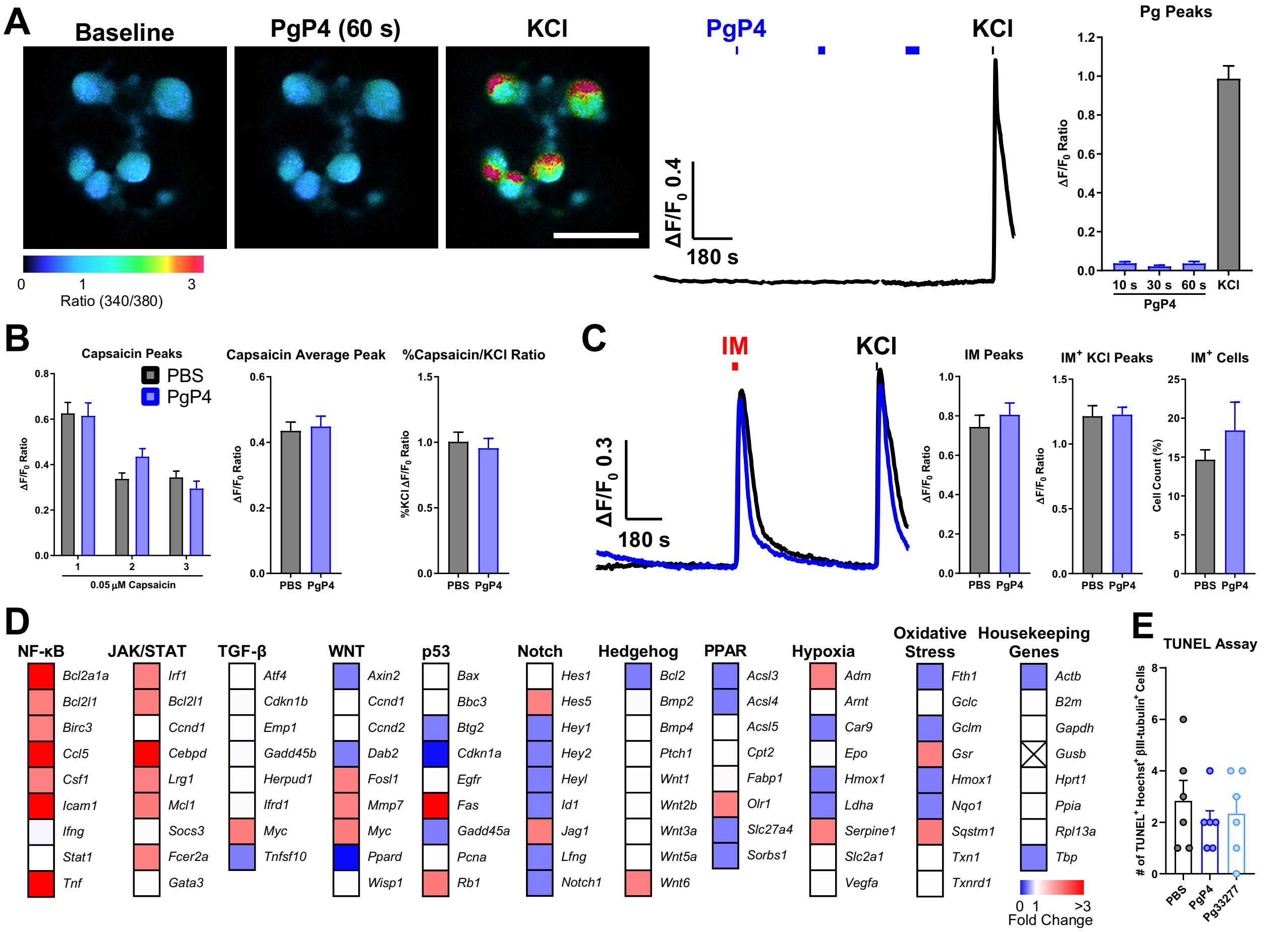

Fig. S2. Pg does not induce acute Ca^{2+} responses in DRG neurons despite upregulating inflammatory marker transcripts, related to Figure 2. (**A**) Representative ratiometric images of Fura-2-labelled DRG neurons (left), representative trace of PgP4-induced calcium response (middle), and comparison of Fura-2 ratio difference among 10-, 30-, and 60-second application of PgP4 (2.5×10^8 CFU/ml) and potassium chloride (KCl, 50 mM) (*n* = 33) from 3 mice (right). Scale bar: 50 μm. (**B**) Comparison of Fura-2 ratio difference of three consecutive 10-second applications of capsaicin (0.05 μM) in 6-hour co-culture with PBS (*n* = 81) or PgP4 (*n* = 79) and DRG neurons from 3 mice (left), average of three capsaicin-induced calcium responses (middle), and ratio of capsaicin-induced calcium response to KCl-induced calcium

responses between PBS (*n* = 81) and PgP4 (*n* = 79) (right). (**C**) Representative traces of inflammatory mediators (IM; a mixture of 10 μM bradykinin, 1 μM prostaglandin E_2, and 1 μM histamine)-induced calcium response in DRG neurons from 6-hour co-culture with PBS (black trace) or PgP4 (blue trace) and comparison of average Fura-2 ratio difference of 30-second application of IM in 6-hour co-culture with PBS (*n* = 46) or PgP4 (*n* = 63) and DRG neurons from 5 mice (left), comparison of KCl-induced calcium responses in IM-responsive (IM^+) cells between PBS (*n* = 46) and PgP4 (*n* = 63) (middle), and comparison of number of IM^+ cells between PBS (*n* = 319) and PgP4 (*n* = 363) (right). (**D**) Heatmap expression of genes involved in several signal transduction pathways measured by qRT-PCR and normalized to GAPDH in 6-hour PgP4-co-cultured DRG neurons compared to PBS (*n* = 1 pooled from 4 mice). (**E**) Number of TUNEL-positive cells were quantified after TUNEL, Hoechst, and βIII-tubulin staining of DRG neurons co-cultured with PBS, Pg33277, or PgP4 for 6 hours. Three random images/dish & two dishes/group were imaged and analyzed. Pairwise Mann-Whitney tests for pre-specified comparisons against PBS controls in (A), (B), (C), and (E). No significant differences were observed. Data are presented as mean ± S.E.M. Pg33277, *Porphyromonas gingivalis* ATCC #33277 strain; PgP4, *Porphyromonas gingivalis* KUMC-P4 strain (clinical isolate); CFU, colony-forming unit.

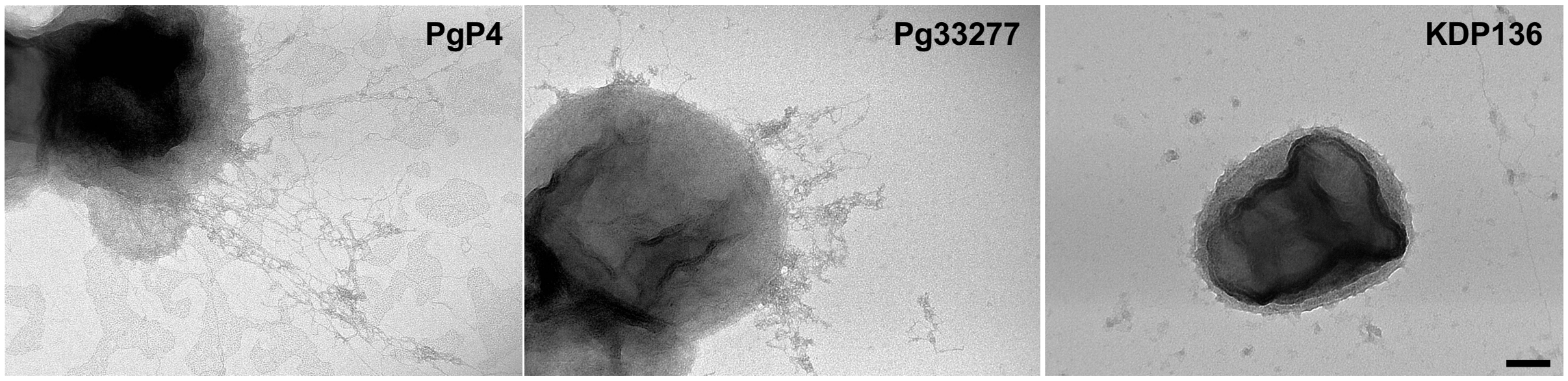

Fig. S3. Gingipain-null mutant, KDP136, lacks fimbriae, related to Figure 3. Transmission electron microscopy of fimbriae in PgP4, Pg33277, and KDP136. Scale bar: 100 nm. PgP4, *Porphyromonas gingivalis* KUMC-P4 strain (clinical isolate); Pg33277, *Porphyromonas gingivalis* ATCC #33277 strain; KDP136, gingipain-null mutant of Pg33277.

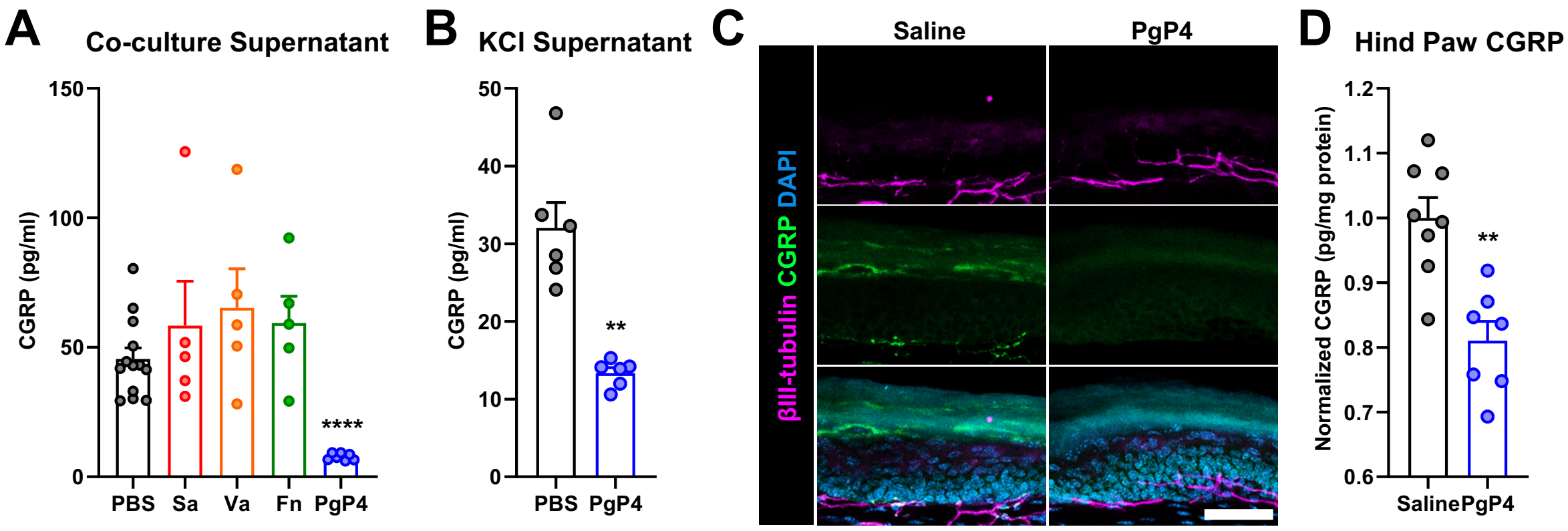

Fig. S4. Pg decreases released CGRP both *in vitro* and *in vivo*, related to Figure 4. (**A**) CGRP levels measured by ELISA in culture supernatants of 6-hour co-culture with PBS (*n* = 13), Sa (*n* = 5), Va (*n* = 5), Fn (*n* = 5), or PgP4 (*n* = 7) and primary cultured DRG neurons from 3 independent experiments ($P < 0.0001$). (**B**) CGRP levels measured by ELISA in culture supernatants of 6-hour co-culture with PBS or PgP4 (*n* = 6/group) and primary cultured DRG neurons with 10-minute KCl treatment at the end from 2 independent experiments ($P = 0.0022$). (**C**) Representative βIII-tubulin- and CGRP-stained images of naïve hind paw skin sections 2 days post-injection of saline or PgP4 from two independent experiments. Scale bar: 50 μm. (**D**) CGRP levels measured by ELISA in naïve hind paw skin sections 2 days post-injection of saline (*n* = 8) or PgP4 (*n* = 7) from 2 independent experiments ($P = 0.0022$). Pairwise Mann-Whitney tests for pre-specified comparisons against PBS controls in (A), (B), and (D). Only significant comparisons are indicated. $^{**}P < 0.01$, $^{****}P < 0.0001$. Data are presented as mean ± S.E.M. Sa, *Staphylococcus aureus*; Va, *Veillonella atypica*; Fn, *Fusobacterium nucleatum*; Pg33277, *Porphyromonas gingivalis* ATCC #33277 strain; PgP4, *Porphyromonas gingivalis* KUMC-P4 strain (clinical isolate); CFU, colony-forming unit; IENF, intraepidermal nerve fiber.
